# Chemoinformatics Analyses of Tau Ligands Reveal Key Molecular Requirements for the Identification of Potential Drug Candidates against Tauopathies

**DOI:** 10.3390/molecules26165039

**Published:** 2021-08-20

**Authors:** Luca Pinzi, Annachiara Tinivella, Giulio Rastelli

**Affiliations:** 1 Department of Life Sciences, University of Modena and Reggio Emilia, Via G. Campi 103/287, 41125 Modena, Italy; luca.pinzi@unimore.it (L.P.); annachiara.tinivella@unimore.it (A.T.); 2 Clinical and Experimental Medicine PhD Program, University of Modena and Reggio Emilia, 41125 Modena, Italy

**Keywords:** Tau, tauopathies, Alzheimer’s Disease, chemoinformatics, ligand-based, molecular descriptors, molecular fragments, drug repurposing, drug design

## Abstract

Tau is a highly soluble protein mainly localized at a cytoplasmic level in the neuronal cells, which plays a crucial role in the regulation of microtubule dynamic stability. Recent studies have demonstrated that several factors, such as hyperphosphorylation or alterations of Tau metabolism, may contribute to the pathological accumulation of protein aggregates, which can result in neuronal death and the onset of a number of neurological disorders called Tauopathies. At present, there are no available therapeutic remedies able to reduce Tau aggregation, nor are there any structural clues or guidelines for the rational identification of compounds preventing the accumulation of protein aggregates. To help identify the structural properties required for anti-Tau aggregation activity, we performed extensive chemoinformatics analyses on a dataset of Tau ligands reported in ChEMBL. The performed analyses allowed us to identify a set of molecular properties that are in common between known active ligands. Moreover, extensive analyses of the fragment composition of reported ligands led to the identification of chemical moieties and fragment combinations prevalent in the more active compounds. Interestingly, many of these fragments were arranged in recurring frameworks, some of which were clearly present in compounds currently under clinical investigation. This work represents the first in-depth chemoinformatics study of the molecular properties, constituting fragments and similarity profiles, of known Tau aggregation inhibitors. The datasets of compounds employed for the analyses, the identified molecular fragments and their combinations are made publicly available as supplementary material.

## 1. Introduction

Tauopathies are a class of heterogeneous neurodegenerative disorders generally characterized by a progressive decline of cognitive functions, change in personality and memory deficit in patients [1,2]. At present, more than twenty neurodegenerative disorders are classified as tauopathies [3,4], the Alzheimer’s Disease (AD) being the most common [5,6]. A common hallmark characterizing these neurological disorders is the abnormal hyperphosphorylation and aggregation of the microtubule-associated protein (MAP) Tau in neurofibrillary tangles (NFTs), especially within the neuronal and *glia* cells [7,8,9]. Tau is a highly soluble protein, ubiquitously expressed in the human brain in six different isoforms [10], which are normally associated with the microtubules (MTs) [11]. From a structural viewpoint, four different regions have been identified in Tau, which belongs to the class of intrinsically disordered proteins (IDPs) [12]. These include: (i) a N-terminal region (NTR) that extends outwards when Tau binds to the MTs, thus regulating its dynamics; (ii) a microtubule-binding region (MBR) hosting the two well-known hexapeptide segments (i.e., PHF6 (VQIVYK) and PHF6* (VQIINK)) involved in the aggregation mechanisms [13,14]; (iii) a proline-rich region (PRR), and; (iv) a C-terminal region (CTR), the latter two being highly conserved in mammals [15]. Under physiological conditions, Tau is involved in the regulation of several signaling processes in cells through the interaction with various binding partners [11,15,16,17]. Importantly, Tau participates in the regulation of microtubules dynamic equilibrium [18] by stimulating tubulin assembly in neuronal cells [19]. The mechanism by which Tau affects the correct physiological function of cells in the brain has yet to be completely elucidated. However, it has been discovered that its physiological function depends on the fine regulation of Tau phosphorylation and dephosphorylation, which is operated by several different proteins in the brain [20]. Several studies have demonstrated that the abnormal hyperphosphorylation, the presence of mutations, and dysregulations in Tau splicing are among the most common causes of tauopathies [15,21,22,23]. In particular, these studies have shown that hyperphosphorylated Tau tends to dissociate from MTs, thus triggering a cascade of events that promote self-aggregation into oligomers forming paired helical (PHF) and straight (SF) filaments. In turn, the formation of such aggregates promotes their assembly into the neurofibrillary tangles typically observed in brains of patients affected by tauopathies [21]. Moreover, the accumulation of hyperphosphorylated Tau aggregates also leads to sequestration of normal Tau and other MAP proteins [21], which are co-responsible for cellular toxicity.

Given the physio-pathological role of this protein in neurodegenerative disorders, several efforts have been made to identify compounds able to disrupt Tau aggregation, and thus restoring the normal physiological function of neurons and *glia* cells [24,25,26]. Notwithstanding, therapeutic remedies currently available for the treatment of tauopathies are limited to amelioration or alleviation of symptoms [2,5]. Based on these premises, the identification of drugs able to prevent or resolve the underlying causes of these neurodegenerative diseases is of utmost importance, especially considering that tauopathies mainly affect elderly people [5,6]. In this respect, several compounds able to modulate Tau aggregation have been reported, most of them acting through non-covalent binding mechanisms [25,27]. Moreover, different approaches have also been pursued to aid in the identification of Tau anti-aggregating agents among natural products [28,29], which represent a rich source of potential drug candidates [30,31]. In addition, inhibitors of protein kinases as GSK-3β, PP_2_A, Fyn and CDK5, which indirectly regulate Tau phosphorylation levels, have been reported [32,33,34]. Furthermore, efforts for the identification of chemical agents that prevent or abrogate Tau aggregation have also been recently reported [32], aurones [35], diamino-phenothiazines [36], rhodanines [27], phenylthiazolyl-hydrazides [27,37], N-Phenylamines, benzothizoles and polyphenols being among the most studied chemical classes [38,39]. However, the majority of the identified ligands resulted from independent screenings, often performed under different experimental conditions. As such, structure-activity relationships (SAR) or pharmacophore requirements useful for the identification of Tau aggregation inhibitors remain largely unknown. In this context, the identification of key structural motifs and molecular properties required to achieve high potency would significantly facilitate drug repurposing and/or the discovery of next generation drug candidates.

Based on these premises, in this work we performed a series of chemoinformatics investigations on ChEMBL [40], which included 2D similarity calculations, molecular fragment analysis, and molecular descriptors evaluation, with the aim of identifying key scaffolds, substructures, and molecular properties responsible for Tau activity. The analyses allowed us to identify a set of chemical fragments, as well as combinations of molecular properties that characterize potent Tau anti-aggregation inhibitors. Such information can be used to assist in the rational design or repositioning of Tau aggregation inhibitors. 

## 2. Materials and Methods

### 2.1. Database Preparation

Tau aggregation inhibitors were collected from the ChEMBL database (accessed on 5 June 2020) [40] and filtered to retain only records with activity annotations related to experiments on isolated protein, expressed in terms of Potency. In particular, only activity records obtained through thioflavin T and S fluorescence assays on the human microtubule-associated protein Tau (UniProt ID: P10636) were considered. Activity records and compounds deriving from different experiments or from cell-based assays were removed. Moreover, multiple activity annotations deriving from different experiments on the same compound were also removed, retaining the one with the best value. This procedure allowed us to obtain a total of 49,284 unique ligands, each associated with a single activity annotation. The molecular structures and activity data of the filtered ligands were finally stored as separated files and made available to readers as supplementary material (see Supporting Information). All steps of ligand dataset generation were performed with the KNIME software (version 4.3.2) [41].

### 2.2. 2D similarity Calculations

The 2D similarity degree of the filtered compounds was first assessed by means of different molecular fingerprints, e.g., MACCS and ECFP4 from the OpenEye python toolkits [42], and AtomPairs and TopologicalTorsion from the RDKit libraries [43]. In particular, all-vs-all similarity estimations were performed for compounds with Potency values below 500 nM (905 compounds), for a total of around 820,000 comparisons. Moreover, the similarity profile of these compounds was also evaluated with respect to those having a reported Potency value higher than 1 µM (47,246 compounds). The 2D similarity was evaluated in terms of the Tanimoto coefficient (Tc), by using default settings [44]. Moreover, the similarity records obtained from the ECFP4 fingerprints-based estimations were further analyzed to evaluate whether one or a selection of active ligands might be considered as representative for the entire population of the most potent Tau aggregation inhibitors considered in the analyses. In particular, extensive analyses were performed to identify a set of compounds among the actives, that were able to retrieve at least 45 ligands with an activity below 500 nM in the curated dataset (corresponding to ~5% of the total), and according to commonly employed similarity thresholds [45]. The selected references were also sequentially combined, up to a maximum of 20 queries *per* combination. This allowed us to identify a set of active ligands, which were able to retrieve the highest number of actives, while keeping the number of inactives low. For each combination, the percentage of retrieved active and inactive ligands was evaluated, and the 20 best performing ones were selected, along with their identified ligands, [45] to be further investigated. Moreover, analysis of the similarity records obtained by ECFP4 fingerprints-based estimations was also performed on the clustered dataset of ligands (see Supporting Information).

### 2.3. Analysis and Comparison of Molecular Descriptors

The molecular properties of the compounds under investigation were first analyzed by using the *QikProp* software available in the Schrödinger suite (release 2020-1) [46], with the default settings. To this aim, the compounds were prepared with *LigPrep* [47] to calculate their ionization states and tautomers potentially present at physiological pH, and to energetically minimize their structure. Then, around 50 molecular properties including drug-likeness and blood-brain-barrier (BBB) permeability were calculated with *QikProp* and compared with the corresponding property ranges of approved drugs.

An especially devised KNIME workflow was also developed to compare molecular properties of the active and inactive compounds in the curated dataset. To this end, 118 different molecular descriptors were first evaluated by using the RDKit nodes implemented in KNIME [43]. Compounds were classified as “*active*” or “*inactive*” according to their reported Potency values. In particular, ligands with Potency values below 500 nM were considered as actives, while different activity thresholds (i.e., ≥1 µM, ≥5 µM, ≥10 µM and ≥20 µM) were considered to classify ligands as “*inactive*”, these values being very often used in chemoinformatics approaches to define inactivity. Statistical details of compounds classified as inactive according to the various thresholds and types of activity are reported in Table S1 (see Supporting Information). Afterwards, the molecular descriptors of the “*active*” and “*inactive*” compounds that provided Pearson Correlation Coefficient (PCC) values higher than 0.95 were removed, and statistical distributions of the others were compared through the use of the one-sided Wilcoxon test [48]. This test has been previously employed to evaluate statistical differences among the properties of populations of ligands in chemoinformatics experiments [49,50], including those that are non-normally distributed. The Bonferroni correction was applied to adjust the significance level (*p*-value) of the analyses for multiple comparisons of the statistical tests [51]. Finally, the molecular descriptors that resulted statistically different in the two populations were further evaluated. This allowed us to highlight relevant differences in the molecular properties of the active and inactive ligands at different thresholds of inactivity.

### 2.4. Molecular Fragment Analyses

An analysis of the molecular fragments characterizing the compounds in the prepared database was also performed. To this aim, the collected compounds were first fragmented by using an *in house* developed python script implemented with the RDKit libraries [43] and the OpenEye toolkits [42]. Different types of fragmentation algorithms were used in the analyses, including BRICS [52], Bemis-Murcko [53] and Recap [54]. Moreover, the Chomp software (version 3.1.1.2—OpenEye) [55] was also used with default settings, as it allows to generate molecular fragments according to the RLF chemical heuristics seek, which allows to break all non-ring and non-resonance single bonds of a given molecule. Then, fragments with a number of atoms outside the range of 5 to 55, and present in less than three molecules were removed. Duplicate structures derived by fragmentation of different compounds were also removed. The number of unique molecular fragments obtained for the active and inactive Tau ligands is reported in Table S2. Afterwards, a workflow implemented in KNIME (version 4.3.2) was devised to identify molecular fragments and their combinations present only, or in common between, active and inactive Tau ligands. Molecular fragments and their combinations generated in these analyses are made available as supplementary material (see Supporting Information).

## 3. Results and Discussion

### 3.1. Dataset Preparation for the Analyses

**Figure 1:**
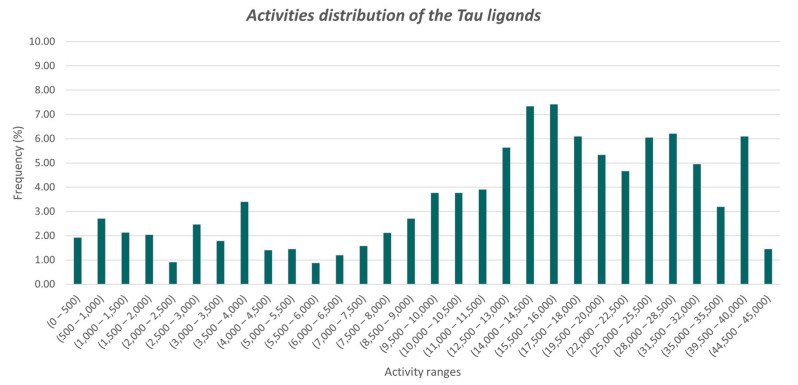
Activity values distribution of known Tau compounds in the curated dataset, each range corresponding to a 500 nM interval. The frequencies are represented by means of their percentage with respect to the total number of activity records (i.e., 49,188). Activity ranges including less than five compounds are not shown.

A dataset of Tau ligands was first generated as detailed in Section 2. The selection of Tau aggregation inhibitors was limited to compounds that were assayed under comparable experimental conditions, by selecting compounds tested with Thioflavin T and S fluorescence assays (see Table S3). Although these assays can be performed *in tandem* with other experiments (e.g., circular dichroism spectroscopy or microscopy, and atomic force microscopy) [35,56,57], in order to circumvent potential false positive readouts, data from the Thioflavin T and S fluorescence experiments provide a rich source of information for chemoinformatics analyses and an appropriate statistical representation of the entire population of Tau chemotypes. Indeed, the number of compounds that were not tested with the Thioflavin T and S fluorescence assays was sensibly lower (see Table S1 in the Supporting Information). Activity data distributions showed that more than 95% of the reported ligands have Potency values above 1 µM (Figure 1), while less than 2% had values below 500 nM. This data is of particular interest, considering that more than 100 chemotypes were identified in the 0–500 nM activity range by visual inspection of their chemical structures.

Of note, some of the molecules in the curated dataset have also been tested in different types of assays, providing comparable results (e.g., see CHEMBL140 and CHEMBL191083 in [58,59]).

### 3.2. Similarity Calculations in the Set of Active Compounds

**Figure 2:**
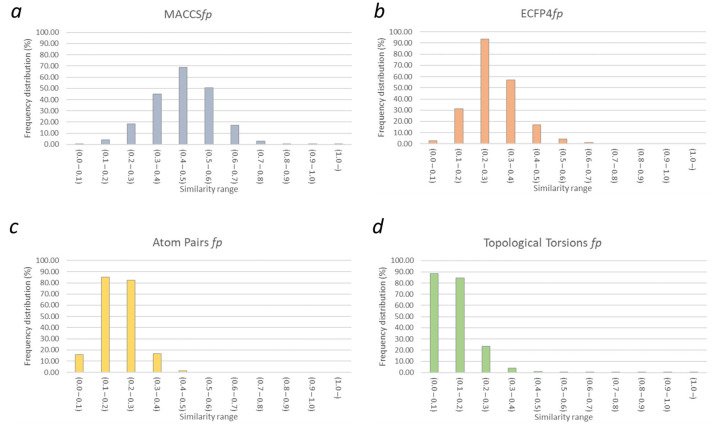
Similarity distributions of Tau ligands in the “*actives*” dataset. Panels (**a**–**d**) show results of the 2D similarities calculated with the MACCS, ECFP4, AtomPairs and TopologicalTorsion fingerprints, respectively.

The similarity profile of compounds in the “*actives*” set (0–500 nM) was evaluated by means of several types of fingerprints, as described above (see Section 2.3). The results, which are shown in Figure 2, highlight an overall low degree of similarity between the compounds of this dataset.

In particular, the similarity analyses showed that the active compounds present high diversity in terms of fragment composition (Figure 2). Indeed, the evaluated Tc values according to MACCS*fp* were largely below the commonly reported similarity threshold (less than 1% of all comparisons). On the contrary, a higher degree of similarity could be observed in their connectivity tables, as more than 38% of the similarities evaluated with ECFP4 fingerprints provided Tc values above 0.3 [45]. Table 1 summarizes the number and percentage of similar pairs identified among active compounds.

According to the performed similarity estimations, different results were observed by using AtomPairs and TopologicalTorsion fingerprints, which evaluate chemical similarity in terms of atom-based and consecutive non-hydrogen bond environments, respectively [61,62]. Indeed, the percentages of similarity revealed by these fingerprints were 2.2% (AtomPairs) and 10.4% (TopologicalTorsion), these values being calculated according to the thresholds for randomness (95% level) suggested in the RDKit documentation [60]. Overall, this analysis highlighted a low degree of similarity between the curated dataset of the Tau active compounds, which mostly originates from their different fragment and chemotype composition. The adopted fingerprints provided a different performance in identifying pairs of similar ligands, the highest numbers of associations being observed for ECFP4*fp* and TopologicalTorsion*fp* also in consensus screenings (Table S4). Of note, ECFP4*fp* showed the best performance in retrieving active compounds (Table 1). However, ECFP4*fp* fingerprints yielded the highest number of comparisons above the selected threshold of similarity (30.8% of the total) when inactive ligands were also included in the similarity analyses (Table 1). Consequently, using ECPF4*fp* in a virtual screening protocol could potentially increase the number of false positives; therefore, the integration with other methods should be carefully evaluated.

The fact that ECFP4*fp* provided the highest number of similarities suggests that this type of fingerprint was able to more efficiently identify common structural patterns among active ligands. Such a feature might be of interest, for example, for the selection of representative queries in a virtual screening. Indeed, an analysis of the ECFP4*fp*-based similarity records showed that CHEMBL1558683 was able to retrieve up to 10% of the active ligands, the statistics of the best performing compounds being reported in Table S5. Different results were observed when inactive compounds were also considered in the similarity analyses. In this case, the highest difference in the percentage of retrieved active vs. inactive ligands was obtained for CHEMBL1555206. Although the best performing compounds were able to retrieve a reasonable number of active ligands, their screening performance was not satisfactory. Indeed, the observed enrichment factors (EF), the area under the curve (AUC) and the BEDROC values were, on average, below the performances desirable in virtual screenings [63,64]. The best AUC and BEDROC values were observed for CHEMBL1512606 (AUC ~0.7) and CHEMBL1377126 (BEDROC ~0.38), respectively. To further evaluate whether the common structural patterns observed in the previous ECFP4*fp*-based analyses might be due to the observed high degree of similarity among small subsets of active ligands, clustering analyses were also performed. In particular, different datasets including active and inactive ligands at different thresholds of inactivity (i.e., ≥1 µM, ≥5 µM, ≥10 µM and ≥20 µM) were first clustered with Canvas (Schrödinger suite 2020-1) [65], as described in the Supporting Information. Then, the ability of the active compounds in each of the generated clusters in retrieving actives and inactives was evaluated. Interesting results were obtained for clusters identified from the datasets of the most potent Tau aggregation inhibitors and ligands with a reported Potency value above 10 µM or 20 µM. Indeed, the majority of them presented at least an active compound showing satisfactory AUC and EF values (Table S6), while poorer prediction performances were obtained for clusters defined by activity thresholds of ≥1 µM and ≥5 µM (Table S6). This result is indicative of the presence of a high number of common structural patterns between small subsets of active ligands, in line with the previously observed chemotype variability.

These results prompted us to also evaluate whether a combination of ligands would be able to more efficiently represent the population of the active compounds in the dataset. To this aim, the similarity data obtained from the ECFP4*fp*-based analyses (see Section 2.2) were further processed, combining the records of the best performing compounds, up to a maximum of twenty ligands *per* combination. For each combination we evaluated the percentages of retrieved active and inactive ligands (see Table 2). The list of reference compounds in the identified combinations is reported in Table S7. The identified combinations of references allowed us to retrieve up to one third of the actives in the curated dataset. The best results were obtained for the combination of 20 queries, both in terms of higher number of actives, and greater difference between the percentages of active and inactive ligands retrieved by the similarity records.

As shown in Table 2 and Figure S1, the rate of retrieved actives per number of queries grows higher than that of the inactives, for combinations of one to seven references. In contrast, the number of actives retrieved by combinations of ten or more queries remained more or less stable. However, results reported in Table S7 showed that the use of combinations of queries still provided poor discriminating performances according to the AUC and BEDROC indexes. Overall, although the use of combinations of queries increased the number of retrieved active compounds, their prediction performances were still not satisfactory. Altogether, results of the similarity analyses did not allow the identification of compounds (or combinations thereof) to be used as representatives of the entire population of actives, or able to discriminate actives from inactives. Instead, results implied the presence of a number of small groups of highly structurally similar ligands in the analyzed dataset, suggesting that 2D similarity estimations alone might not be an ideal method to perform, for example, virtual screening on this target.

### 3.3. Analysis and Comparison of Molecular Descriptors

The compounds were then evaluated for their drug-like properties and blood-brain-barrier (BBB) permeability. This analysis was performed by means of the *QikProp* software available in the Schrödinger suite (release 2020-1) [46,47]. The results showed that the majority of the compounds present good drug-like properties and are able to cross the BBB, such prediction being especially true for compounds with activity below 500 nM. The results of this analysis are reported in Table S8.

**Figure 3:**
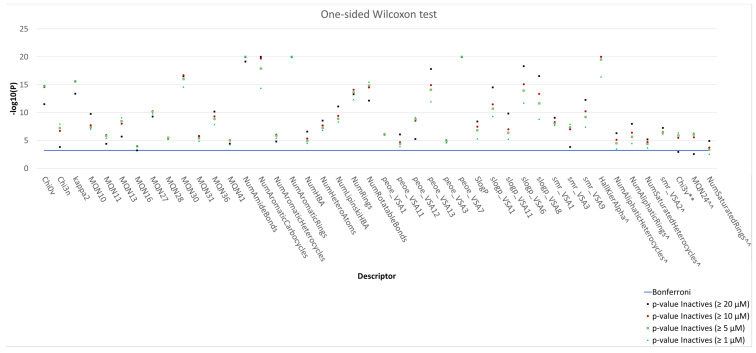
Distribution plot of statistically different molecular descriptors. Each distribution is represented by means of its negative logarithmic *p*-value of the one-sided Wilcoxon test. The Bonferroni threshold, which is displayed as a blue line, highlights the adjusted significance level (*p*-value) for multiple comparisons at statistical tests. Molecular descriptors marked with “^” are those whose values are on average lower for the inactive compounds with respect to the actives. Molecular descriptors marked as “**” present values that are, on average, higher for the active compounds, with respect to inactives at 1 µM, 5 µM and 10 µM activity thresholds. Molecular descriptors that are marked with “^^” present values that are, on average, higher for the active compounds, with respect to inactives at 5 µM, 10 µM and 20 µM activity thresholds.

Extensive in silico analyses were then performed to evaluate whether the active and inactive compounds present statistically different molecular properties. The analyses were performed on different populations of inactive ligands, i.e., by considering ≥1 µM, ≥5 µM, ≥10 µM and ≥20 µM inactivity thresholds, and by using the 118 molecular descriptors implemented in the “RDKit Descriptor Calculation” node available in KNIME. Molecular descriptors with PCC ≥ 0.95 were removed to avoid potential inter-correlation issues. Then, a statistical comparison of the remaining molecular descriptors was performed through a one-sided Wilcoxon test, to evaluate whether the mean values in the actives and inactives datasets were statistically different. The results, shown in Figure 3 and Figure S2, demonstrate that 41 of the calculated molecular descriptors provided statistically different results (see Table S9), although none of them alone was able to clearly separate actives from inactives (see Table S10). These results are likely due to the high molecular diversity observed in the dataset.

Interestingly, these analyses allowed us to delineate a set of molecular properties that should be present in active compounds.

In particular, the introduction of aryl-substituted H-bond acceptors is generally favored. Indeed, the *NumAmideBonds* and *NumHBA* descriptors were on average higher for the active compounds. Moreover, active compounds had a higher number of aromatic and heteroaromatic rings compared to the inactives (on average from 2 to 4 and up to 2, respectively). Also, the presence of aliphatic moieties is associated with inactivity, as highlighted by the lower values of the *NumAliphaticHeterocycles*, *NumAliphaticRings* and *NumSaturatedHeterocycles* molecular descriptors in the active compounds. Consistently, the *NumSaturatedRings* of inactive compounds was lower at 5 µM, 10 µM and 20 µM inactivity thresholds. Together, these results suggest that an increase of the aliphatic character of the molecules result in a decrease of activity. Moreover, it also suggests that aromatic compounds able to establish π–π interactions may more efficiently interact with Tau. The active compounds had, on average, higher values of *logP* (Table S10). Active and inactive compounds also differ for their atomic valence connectivity indexes, which tend to be higher for the former class (e.g., *Chi0v, Chi3n* and *Kappa 2*) [66]. Moreover, higher values of MOE-type descriptors related to the surface area with partial charges (e.g., *PEOE_VSA*_1,3,7,11,12,13_), molar refractivity (e.g., *SMR_VSA*_1,3,9_) and *LogP* (e.g., *SlogP_VSA*_1,6,8,11_) were also observed for the more active compounds [66,67]. Finally, topological descriptors based on the count of simple structural features, i.e., cyclic divalent nodes (MQN30) and 6-membered rings (MQN36) [68], were higher for the active compounds. In conclusion, the analysis of molecular descriptors provided some hints on molecular properties that active compounds should possess, which could be useful for drug design.

### 3.4. Analysis of Molecular Fragments

**Figure 4:**
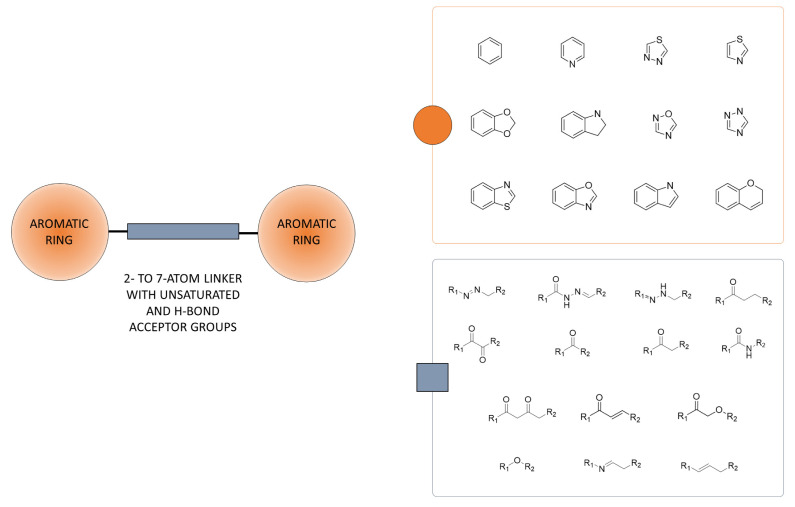
General molecular framework identified by visual inspection of the more frequently observed combinations of fragments in the active compounds. A list of the top-100 combinations observed in the more active ligands is reported in Sections *A* and *B* of Table S15.

An analysis of the molecular fragment composition for compounds in the curated dataset was also performed. Fragmentation was performed by means of five different algorithms. This allowed us to exhaustively identify chemical moieties and functional groups present in Tau active and/or inactive compounds at different thresholds of inactivity. In particular, we were able to identify 38 chemical moieties exclusively present in the active ligands (Table S11), and 70,330, 63,678, 57,407 and 25,637 molecular fragments present only in the inactives at 1 µM, 5 µM, 10 µM and 20 µM thresholds of inactivity, respectively. Statistics related to the identified fragments are reported in Table S12. Visual inspection of the molecular fragments of active compounds revealed the presence of at least one hydrogen bond acceptor group. Moreover, around 50% of them had also one or more aromatic or heteroaromatic ring(s), often bonded together or through carbonyl and/or linear alkenes linkers. A high number of fragments in common between the active and inactive sets have also been detected. However, also in this case, aromatic and heteroaromatic groups and fragments bearing H-bond acceptors were generally prevalent in the active compounds (Table S12). On the contrary, molecular fragments with hydrophilic rings, such as piperazine and morpholine, or saturated rings, were more present in the inactive compounds. A list of the more frequently observed molecular fragments is reported in Table S13, along with their percentages of occurrence in the active and inactive sets. Altogether, these results suggest that aromatic/heteroaromatic fragments and H-bond acceptors are expected to contribute favorably to activity, as opposed to hydrophilic or saturated chemical moieties. The complete list of molecular fragments identified in the analysis is provided as supplementary material (see Supporting Information). Interestingly, we also found that certain combinations of molecular fragments are especially present in the active dataset (Table S14). These include the combination of 2 or 3 molecular fragments, for example the phenyl and carbonyl groups with fragments bearing other H-bond acceptors, which were present in more than 32% and 16% of the active compounds, respectively (see Table S14, sections A and B). The combinations of molecular fragments identified in active compounds are provided as supplementary material (see Supporting Information). Of note, visual inspection of the more frequent fragment combinations revealed that active compounds are often organized into molecular frameworks composed by two aromatic or heteroaromatic rings separated by different 2- to 7-atom linkers. This latter moiety is very often decorated with H-bond acceptors or may consist of rigid linear unsaturated functional groups (Figure 4).

Notably, the identified framework is present in some of the already reported Tau-based drug candidates currently undergoing clinical trials (e.g., xalsalate and curcumin) (Figure S3) [69,70], as well as in the natural compounds xanthohumol and licochalcone A that act as Tau aggregation inhibitors [70,71] (Figure S3). The latter chemotype is currently under evaluation on several targets related to neurodegenerative diseases, including monoaminoxidase B, αβ-amyloid and α-sinuclein [72,73,74]. These findings further strengthen the potential use of molecular fragment combinations based on this framework as starting points for the development of novel Tau aggregation inhibitors.

## 4. Conclusions

In this work, we have reported the first extensive chemoinformatics study on Tau ligands that systematically analyzed the similarity profiles, molecular properties and chemical fragments of a large dataset of compounds with known activity on Tau aggregation. In particular, similarity estimations were performed by means of different types of fingerprints, identifying a high degree of structural diversity. Comparison of the molecular properties of active and inactive compounds confirmed the presence of significant structural diversity and allowed us to outline a set of molecular properties that compounds should possess in order to display high potency. A comparative analysis of the molecular fragments of Tau ligands corroborated the results obtained with the molecular descriptors and brought attention to the need for aromatic/heteroaromatic rings and hydrogen bond acceptors, as opposed to hydrophilic or saturated chemical moieties. Notably, an analysis of fragment combinations showed that active compounds are often organized into molecular frameworks composed by two aromatic or heteroaromatic rings separated by different 2- to 7-atom linkers, the latter moiety being very often decorated with H-bond acceptors or possibly consisting of rigid linear unsaturated functional groups. Altogether, the results of this study showed the possibility to identify a set of molecular properties and fragment combinations that can be useful for *de novo* drug design or drug repurposing campaigns.

## Figures and Tables

**Table 1:** Results of the similarity estimations performed in the curated dataset of Tau ligands. The similarity estimations were performed in the “*actives*” (0–500 nM) and “*inactives*” (≥1 µM) datasets.

Type of Fingerprints ^1^	Number of Identified Pairs (Actives) ^2^	% of Identified Pairs (Actives) ^3^	Number of Identified Pairs (Inactives) ^2^	% of Identified Pairs (Inactives) ^3^
MACCS*fp*	1859	0.5	27,604	0.1
ECFP4*fp*	159,125	38.8	13,460,261	30.8
AtomPairs*fp*	9093	2.2	488,407	1.1
TopologicalTorsion*fp*	42,477	10.4	2,670,815	6.1

^1^ Similarity thresholds were taken from [45] for MACCS and ECFP4, and from [60] for AtomPairs and TopologicalTorsion fingerprints. ^2^ The total numbers of active and inactive compounds considered in the analyses are 905 and 47,246, respectively. ^3^ The total number of comparisons performed by considering only active compounds are 409,965, which became 43,733,220 by including also inactive ligands.

**Table 2:** Percentages of active and inactive ligands retrieved by the best performing combinations of reference compounds, according to the ECFP4 similarity estimations.

Number of Queries *per* Combination	% of Actives (≤500 nM) ^1^	% of Inactives (≥1 µM) ^1^	Difference between the % of *Actives* and *Inactives* ^2^
1	10.8 (98)	3.2 (1568)	7.6 (16)
2	18.1 (164)	5.1 (2465)	13.0 (15)
3	22.4 (203)	6.6 (3189)	15.8 (16)
4	24.2 (219)	6.7 (3238)	17.5 (15)
5	28.3 (256)	9.5 (4591)	18.8 (18)
6	30.1 (272)	10.5 (5074)	19.6 (19)
7	32.7 (296)	12.3 (5944)	20.4 (20)
8	32.2 (291)	12.1 (5847)	20.1 (20)
9	33.7 (305)	13.6 (6572)	20.1 (22)
10	33.8 (306)	13.9 (6717)	19.9 (22)
11	32.9 (298)	12.9 (6234)	20.0 (21)
12	33.7 (305)	13.3 (6427)	20.4 (21)
13	35.0 (317)	14.3 (6910)	20.7 (22)
14	35.0 (317)	14.4 (6959)	20.6 (22)
15	35.1 (318)	14.5 (7007)	20.6 (22)
16	35.7 (323)	14.8 (7152)	20.9 (22)
17	36.8 (333)	15.7 (7587)	21.1 (23)
18	36.8 (333)	15.7 (7587)	21.1 (23)
19	36.8 (333)	15.6 (7539)	21.2 (23)
20	37.1 (336)	15.9 (7684)	21.2 (23)

^1^ The number of compounds retrieved by the combination of references is reported in round brackets. ^2^ The number of inactives *per* active compound is reported in round brackets. The total numbers of active and inactive compounds are 905 and 47,246, respectively.

## Data Availability

Not applicable.

## Supplementary Materials

The following are available online, Figure S1: Percentages of active and inactive compounds retrieved by means of the selected query combinations.; Figure S2: Distribution plots of the molecular descriptors compared in the analyses.; Figure S3: Chemical structures of the xalsalate, curcumin, xanthohumol and licochalcone A known Tau aggregation inhibitors.; Table S1: Number of ligands according to different activity thresholds and type.; Table S2: Number of unique molecular fragments identified from the analyses of the active and inactive datasets.; Table S3: Assay types and description related to the compounds considered in this work.; Table S4: Statistics related to the combinations of the different fingerprints employed in the similarity estimations.; Table S5: Percentages of actives and inactive ligands retrieved by the best performing queries, according to the ECFP4*fp* similarity estimations.; Table S6: Best-three performing queries identified for each cluster in the different datasets of ligands.; Table S7: Results of the ECFP4*fp*-based similarity screenings obtained for the best performing set of queries per combination.; Table S8: Percentages of Tau aggregation inhibitors that present good drug-like properties according to commonly recommended values, as evaluated with QikProp (Schrödinger 2020-1).; Table S9: Mean (±Standard deviation), and 10th and 90th percentiles evaluated for the molecular descriptors that emerged as different for the active and inactive compounds.; Table S10: Percentage of compounds with molecular descriptors within the 10th and 90th percentile thresholds of the active compounds.; Table S11: Molecular fragments observed exclusively in the active compounds.; Table S12: Statistics related to molecular fragments identified in the active and inactive datasets.; Table S13: Molecular fragments that provided the larger difference in their prevalence across the active and inactive classes.; Table S14: Combinations of fragments more frequently observed in the active ligands, according to percentages of occurrence.; Ligands clustering; The datasets of compounds employed for the analyses, the molecular fragments and their combinations identified are provided as supplementary material.

## Sample Availability

Samples are available from the authors.
