# Supplementary material for: Chemoinformatics Analyses of Tau Ligands Reveal Key Molecular Requirements for the Identification of Potential Drug Candidates against Tauopathies
Source: Molecules. 2021 Aug 20;26(16):5039. doi: 10.3390/molecules26165039 (PMC8400687; doi:10.3390/molecules26165039)
Supplement: Supplementary file 1 [file molecules-26-05039-s001.zip › molecules-26-05039-s001/molecules-1308144-supplementary.pdf]

molecules

Article

Chemoinformatics Analyses of Tau Ligands Reveal Key Molecular Requirements for the Identification of Potential Drug Candidates against Tauopathies

Luca Pinzi [1], Annachiara Tinivella [1,2] and Giulio Rastelli [1,*]

[1] Department of Life Sciences, University of Modena and Reggio Emilia, Via G. Campi 103/287, 41125 Modena, Italy; luca.pinzi@unimore.it (L.P.); annachiara.tinivella@unimore.it (A.T.)
[2] Clinical and Experimental Medicine, PhD Program, University of Modena and Reggio Emilia, Modena, Italy
* Correspondence: giulio.rastelli@unimore.it; Tel.: +39-059-2058564

SUPPORTING INFORMATION

TABLE OF CONTENTS

Figures

Figure S1 .... S3
Figure S2 .... S4
Figure S3 .... S5

Tables

Table S1 .... S6
Table S2 .... S7
Table S3 .... S8
Table S4 .... S9
Table S5 .... S8
Table S6 .... S13
Table S7 .... S25
Table S8 .... S26
Table S9 .... S27
Table S10 .... S28
Table S11 .... S30
Table S12 .... S32
Table S13 .... S33
Table S14 .... S34
Table S15 .... S36

Ligands clustering S40

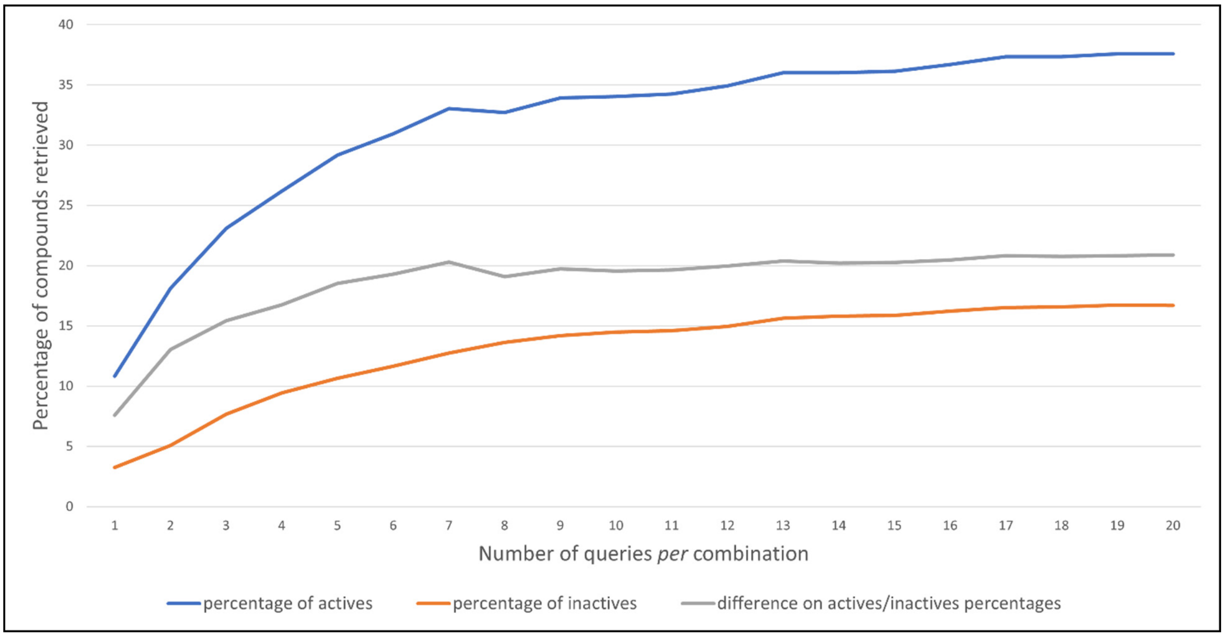

Figure S1. Percentages of active and inactive compounds retrieved by means of the selected query combinations. The rates of actives and inactives are depicted with blue and orange lines, respectively. Thegrey line reports the difference between active and inactive percentages. As shown in the picture, the difference between actives and inactives grow up to the combination of 7 queries, after which it remains stable at around 20%. Considering that the population of actives (905 ligands) and inactives (47246 ligands) are different, a high number of queries in the screening would provide results more populated by inactive compounds.

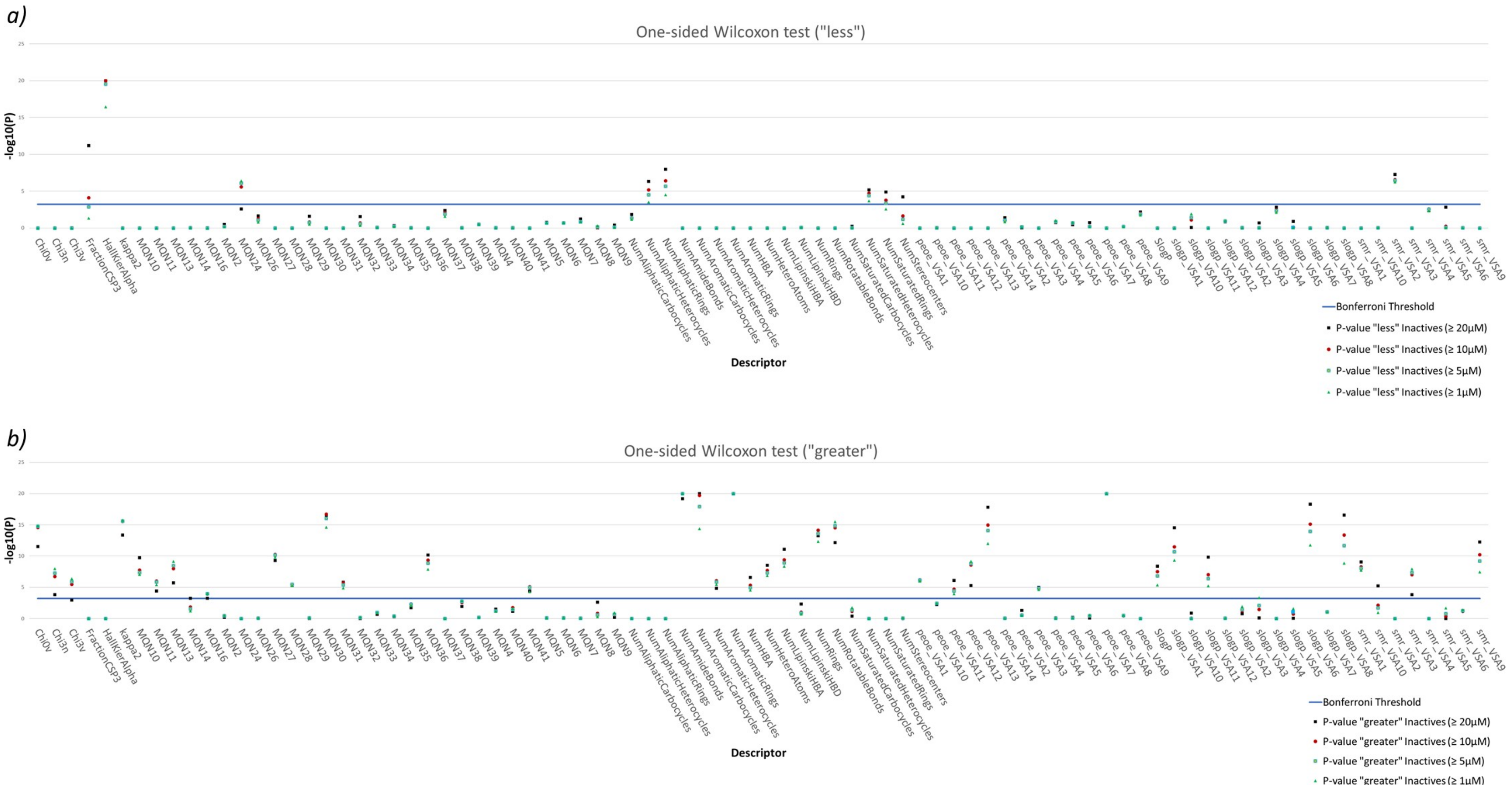

Figure S2. Distribution plots of the molecular descriptors compared in the analyses. Each distribution is represented by means of its negative logarithmic *P-value* of the one-sided Wilcoxon test. Panels ***a*** and ***b*** report the results of the one-sided Wilcoxon test as the mean values of the actives resulted inferior ("less") and superior ("greater") compared to those of the inactives at different thresholds of inactivity, respectively. The Bonferroni threshold, which is displayed as a blue line, highlights the adjusted significance level (*P-value*) for multiple comparisons at statistical tests.

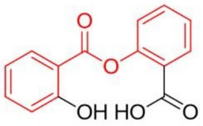

Xalsalate

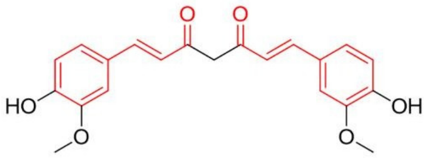

Curcumin

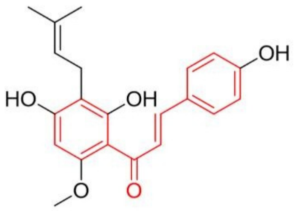

Xanthohumol

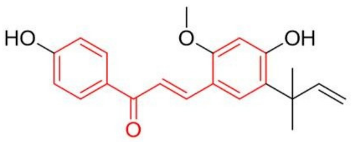

Licochalcone A

Figure S3. Chemical structures of the xalsalate, curcumin, xanthohumol and licochalcone A known Tauaggregation inhibitors. The molecular framework identified in the fragment analysis is highlighted in red.

Table S1. Number of ligands according to different activity thresholds and type.

Copyright: © 2021 by the authors. Licensee MDPI, Basel, Switzerland. This article is an open access article distributed under the terms and conditions of the Creative Commons Attribution (CC BY) license (http://creativecommons.org/licenses/by/4.0/).

**Compounds**	**≤ 500 nM**	**≥ 1 μM**	**≥ 5 μM**	**≥ 10 μM**	**≥ 20 μM**
Potency	905	47192	40286	35412	16011
IC_{50}	7	19	13	10	6
K_i	34	4	0	0	0

Table S2. Number of unique molecular fragments identified from the analyses of the active and inactivedatasets.

	Number of fragments[1]	*Number of fragments (≥3 molecules)*[2]	*Number of fragments (≥10 molecules)*[3]
Actives (≤500nM)	17544	1918	219
Inactives (≥1μM)	316532	84917	9023
Inactives (≥5μM)	297486	77220	7433
Inactives (≥10μM)	279808	69460	6369
Inactives (≥20μM)	190079	31537	2122

Note: The total number of active compounds considered in the analysis is 905. Numbers were 47246, 40325, 35448, and 16034 for the ≥1μM, ≥5μM ≥10μM and ≥20μM activity thresholds, respectively. [1] molecular fragments present in at least one compound from the BRICS, Bemis-Murcko, Recap and Chomp analyses. [2] molecular fragments present in at least three compounds from the BRICS, Bemis-Murcko, Recap and Chompanalyses. [3] molecular fragments present in at least ten compounds from the BRICS, Bemis-Murcko, Recap and Chomp analyses.

Table S3. Assay types and description related to the compounds considered in this work. Further detailson the assays can be found at https://pubchem.ncbi.nlm.nih.gov/bioassay/1460.

Assay ChEMBL ID	*Assay Description*
CHEMBL1614421	PUBCHEM_BIOASSAY: qHTS for Inhibitors of Tau Fibril Formation, Thioflavin TBinding. (Class of assay: confirmatory) [Related pubchem assays: 596]
CHEMBL1613828	PUBCHEM_BIOASSAY: Tau ThS binding assay. (Class of assay: confirmatory) [Related pubchem assays: 596]
CHEMBL1614365	PUBCHEM_BIOASSAY: Confirmation Concentration-Response Assay for Inhibitors of Tau Fibril Formation, Thioflavin T Binding. (Class of assay: confirmatory) [Related pubchem assays: 1460]

Table S4. Statistics related to the combinations of the different fingerprints employed in the similarity estimations. The similarity analyses were performed by considering all compounds within the 0-500nM activity range. Ligands were considered as inactive if their activity was higher than 1μM.

Type of Fingerprints [1]	Number of pairs identified(actives) [1]	% of pairsidentified(actives) [3]	Number ofpairs identified (inactives) [2]	% of pairsidentified (inactives) [3]
MACCS*fp* ∩ ECFP4*fp*	1838	0.44	25817	0.06
MACCS*fp* ∩ AtomPairs*fp*	1662	0.41	17272	0.04
MACCS*fp* ∩ TopologicalTorsion*fp*	1774	0.43	21513	0.05
ECFP4*fp* ∩ AtomPairs*fp*	7831	1.91	369070	0.84
ECFP4*fp* ∩ TopologicalTorsion*fp*	34052	8.31	1938487	4.43
AtomPairs*fp* ∩ TopologicalTorsion*fp*	6497	1.59	269847	0.62
MACCS*fp* ∩ ECFP4*fp* ∩ AtomPairs*fp*	1660	0.40	16811	0.04
MACCS*fp* ∩ ECFP4*fp* ∩ TopologicalTorsion*fp*	1770	0.43	21081	0.05
MACCS*fp* ∩ AtomPairs*fp* ∩ TopologicalTorsion*fp*	1648	0.40	15795	0.04
ECFP4*fp* ∩ AtomPairs*fp* ∩ TopologicalTorsion*fp*	6033	1.47	229966	0.53
MACCS*fp* ∩ ECFP4*fp* ∩ AtomPairs*fp* ∩ TopologicalTorsion*fp*	1647	0.40	15565	0.03

[1] Similarity thresholds were retrieved from (doi: 10.12688/f1000research.8357.2) for MACCS and ECFP4, and from (http://rdkit.blogspot.com/2013/10/fingerprint-thresholds.html) for the AtomPairs and TopologicalTorsion fingerprints. [2] The total numbers of active and inactive compounds considered in the analyses are 905 and 47246, respectively. [3] The total number of comparisons performed by considering only active compounds is 409965, which became 43733220 by including inactive ligands.

Table S5. Percentages of actives and inactive ligands retrieved by the best performing queries, according to the ECFP4*fp* similarity estimations.The queries that provided the best performances in terms of % of retrieved actives and differences between the active and inactive ligands, respectively are shown in red and blue, respectively. .

Query	*% of Actives (≤ 500nM)*[1]	*% of Inactive (≥ 1μM)*[1]	*difference % Actives vs Inactives*[2]	*AUC*	*EF (1%)*	*EF (5%)*	*EF (5%)*	*BEDROC*
CHEMBL1558683	10.8 (98)	3.2 (1568)	7.6 (16)	0.675	3	2.429	2.036	0.185
CHEMBL1544910	10.7 (97)	3.5 (1679)	7.2 (17)	0.543	4.069	1.852	1.646	0.144
CHEMBL1442023	10.6 (96)	2.8 (1367)	7.8 (14)	0.678	4.064	4.531	2.488	0.293
CHEMBL1355273	10.5 (95)	2.8 (1364)	7.7 (14)	0.652	4.095	3.366	2.84	0.27
CHEMBL1476765	10.5 (95)	2.9 (1389)	7.6 (15)	0.647	5.207	3.124	3.25	0.285
CHEMBL1555206	10.2 (92)	2.1 (1029)	8.1 (11)	0.618	5.077	3.42	2.372	0.297
CHEMBL1354557	10.2 (92)	3.1 (1501)	7.1 (16)	0.616	4.329	2.597	1.732	0.189
CHEMBL1431458	9.5 (86)	4 (1914)	5.5 (22)	0.554	2.326	0.93	1.279	0.092
CHEMBL1405215	9.2 (83)	2 (975)	7.2 (12)	0.704	4.635	3.608	2.646	0.294
CHEMBL1607810	9.2 (83)	2.1 (991)	7.1 (12)	0.676	8.234	3.115	2.636	0.308
CHEMBL1572407	8.4 (76)	2 (949)	6.4 (13)	0.531	3.678	1.556	1.048	0.145
CHEMBL1592536	8.4 (76)	2.1 (1000)	6.3 (13)	0.583	5.148	2.884	1.966	0.241
CHEMBL1530889	8.2 (74)	1.4 (694)	6.8 (9)	0.616	6.486	3.193	2.291	0.327
CHEMBL1591502	8.2 (74)	1.9 (919)	6.3 (12)	0.593	4.026	1.61	1.476	0.162
CHEMBL3194159	8.1 (73)	3.1 (1477)	5 (20)	0.541	2.654	1.633	1.096	0.111
CHEMBL1495007	8 (72)	1.6 (775)	6.4 (11)	0.664	2.614	3.283	2.906	0.283
CHEMBL1330086	8 (72)	1.7 (838)	6.3 (12)	0.646	5.056	2.748	2.639	0.263
CHEMBL1532527	7.8 (71)	1.1 (524)	6.7 (7)	0.594	1.397	1.955	2.235	0.259
CHEMBL1577023	7.8 (71)	1.8 (846)	6 (12)	0.683	5.166	3.931	2.527	0.31
CHEMBL1401843	7.7 (70)	1.4 (675)	6.3 (10)	0.613	5.321	2.241	1.987	0.245
CHEMBL1438558	7.7 (70)	2 (958)	5.7 (14)	0.675	5.34	2.542	2.709	0.253
CHEMBL1399653	7.7 (70)	2.2 (1056)	5.5 (15)	0.661	4.021	3.386	2.847	0.27
CHEMBL1564465	7.6 (69)	2.2 (1082)	5.4 (16)	0.573	5.56	1.726	1.726	0.171
CHEMBL1417596	7.5 (68)	1.3 (631)	6.2 (9)	0.615	1.468	2.643	2.203	0.253
CHEMBL1549072	7.4 (67)	1.7 (839)	5.7 (13)	0.665	6.761	3.528	2.823	0.29
CHEMBL1476630	7.3 (66)	2.1 (1031)	5.2 (16)	0.534	1.511	0.604	1.36	0.097

CHEMBL1481415	7.1 (64)	1.4 (698)	5.7 (11)	0.684	5.953	3.053	2.319	0.283
CHEMBL1315383	7 (63)	1.5 (715)	5.5 (11)	0.672	3.087	2.533	2.217	0.247
CHEMBL1553703	7 (63)	1.8 (859)	5.2 (14)	0.659	4.39	1.557	1.574	0.169
CHEMBL1515009	6.9 (62)	1.2 (565)	5.7 (9)	0.608	2.889	1.896	1.445	0.194
CHEMBL1572149	6.6 (60)	1.3 (619)	5.3 (10)	0.552	3.233	1.997	1.997	0.237
CHEMBL1433634	6.6 (60)	1.5 (746)	5.1 (12)	0.591	4.478	1.966	1.658	0.179
CHEMBL1553843	6.5 (59)	1.5 (711)	5 (12)	0.636	3.263	1.339	1.356	0.14
CHEMBL1425944	6.5 (59)	1.7 (833)	4.8 (14)	0.61	1.68	2.016	2.184	0.161
CHEMBL1593426	6.4 (58)	1.5 (702)	4.9 (12)	0.683	1.638	3.103	2.759	0.283
CHEMBL1328302	6.4 (58)	2.2 (1042)	4.2 (18)	0.575	3.448	2.069	1.379	0.139
CHEMBL1368354	6.3 (57)	1 (495)	5.3 (9)	0.641	3.228	1.383	1.556	0.212
CHEMBL1395944	6.3 (57)	1.1 (549)	5.2 (10)	0.681	3.038	3.087	2.789	0.307
CHEMBL1572603	6.3 (57)	1.2 (596)	5.1 (11)	0.573	4.91	2.083	1.389	0.19
CHEMBL1339146	6.3 (57)	1.5 (703)	4.8 (12)	0.555	3.333	1.754	2.456	0.21
CHEMBL260326	6.2 (56)	1.3 (608)	4.9 (11)	0.599	5.082	2.441	1.947	0.25
CHEMBL1364914	6.2 (56)	1.3 (644)	4.9 (12)	0.633	7.143	2.857	2.679	0.312
CHEMBL1488396	6.2 (56)	1.5 (709)	4.7 (13)	0.533	3.415	0.701	1.774	0.145
CHEMBL1535640	6.2 (56)	1.9 (908)	4.3 (16)	0.581	3.443	3.162	2.13	0.195
CHEMBL1511385	6.1 (55)	1.1 (552)	5 (10)	0.688	3.153	3.204	2.533	0.307
CHEMBL1567897	6.1 (55)	1.3 (608)	4.8 (11)	0.691	3.444	2.836	2.699	0.267
CHEMBL1552096	6.1 (55)	1.3 (639)	4.8 (12)	0.613	3.605	1.803	2.163	0.22
CHEMBL1347420	6.1 (55)	1.4 (690)	4.7 (13)	0.575	3.386	1.782	1.084	0.152
CHEMBL1318968	6.1 (55)	1.4 (694)	4.7 (13)	0.676	6.809	2.867	2.36	0.287
CHEMBL1575808	6.1 (55)	1.6 (796)	4.5 (15)	0.572	6.877	3.238	1.979	0.245
CHEMBL1476840	6 (54)	0.9 (427)	5.1 (8)	0.667	7.126	2.85	1.818	0.335
CHEMBL1472767	6 (54)	2.4 (1137)	3.6 (21)	0.469	1.838	0.735	0.735	0.069
CHEMBL1377126	5.9 (53)	0.9 (416)	5 (8)	0.592	5.309	4.056	2.824	0.378
CHEMBL1408839	5.9 (53)	1 (494)	4.9 (9)	0.572	1.72	1.474	1.877	0.178
CHEMBL1418237	5.9 (53)	1.2 (576)	4.7 (11)	0.641	6.782	2.967	2.637	0.317
CHEMBL1553528	5.9 (53)	1.4 (657)	4.5 (12)	0.685	6.698	4.093	3.019	0.339
CHEMBL1436651	5.9 (53)	2 (982)	3.9 (19)	0.515	1.775	1.127	1.127	0.103
CHEMBL1531961	5.9 (53)	3 (1434)	2.9 (27)	0.568	1.87	1.87	1.506	0.107
CHEMBL1451292	5.7 (52)	0.8 (373)	4.9 (7)	0.655	4.904	3.344	2.091	0.358
CHEMBL1512013	5.7 (52)	1.3 (614)	4.4 (12)	0.604	3.659	2.637	2.676	0.241

CHEMBL1612392	5.7 (52)	1.3 (634)	4.4 (12)	0.607	1.885	1.885	2.677	0.217
CHEMBL1362468	5.7 (52)	2 (970)	3.7 (19)	0.618	1.787	1.134	0.954	0.102
CHEMBL1554186	5.7 (52)	2.1 (1012)	3.6 (20)	0.535	7.441	2.652	1.53	0.17
CHEMBL1535192	5.7 (52)	2.7 (1295)	3 (25)	0.592	5.551	3.048	1.919	0.173
CHEMBL1495115	5.6 (51)	1.1 (537)	4.5 (11)	0.584	1.922	1.537	2.15	0.186
CHEMBL1410048	5.6 (51)	1.3 (618)	4.3 (12)	0.514	3.748	1.157	0.979	0.136
CHEMBL1530146	5.6 (51)	2 (954)	3.6 (19)	0.594	3.583	1.159	1.171	0.115
CHEMBL404214	5.6 (51)	2.7 (1286)	2.9 (25)	0.483	1.873	0.783	0.587	0.058
CHEMBL1518345	5.5 (50)	0.9 (427)	4.6 (9)	0.565	1.908	1.192	1.192	0.152
CHEMBL1318911	5.5 (50)	1.5 (745)	4 (15)	0.512	1.988	1.988	1.192	0.142
CHEMBL1366547	5.5 (50)	1.9 (915)	3.6 (18)	0.586	3.86	1.969	1.393	0.153
CHEMBL1321724	5.5 (50)	2 (977)	3.5 (20)	0.577	1.867	0.395	0.399	0.056
CHEMBL1429397	5.4 (49)	1 (475)	4.4 (10)	0.597	5.347	2.772	1.816	0.268
CHEMBL1512606	5.4 (49)	1 (477)	4.4 (10)	0.707	3.578	2.783	2.43	0.297
CHEMBL1613377	5.4 (49)	1 (500)	4.4 (10)	0.573	3.735	1.601	1.426	0.186
CHEMBL1474025	5.4 (49)	1.1 (542)	4.3 (11)	0.602	2.01	2.814	2.211	0.234
CHEMBL1545593	5.4 (49)	1.5 (741)	3.9 (15)	0.599	2.015	2.418	1.633	0.168
CHEMBL3194482	5.4 (49)	2.9 (1408)	2.5 (29)	0.528	1.982	0.815	1.018	0.084
CHEMBL1583382	5.3 (48)	1.1 (516)	4.2 (11)	0.514	3.917	2.431	1.855	0.217
CHEMBL1449627	5.3 (48)	1.2 (574)	4.1 (12)	0.637	3.702	2.025	2.468	0.221
CHEMBL1421876	5.3 (48)	3.5 (1698)	1.8 (35)	0.496	4.042	2.067	1.455	0.128
CHEMBL1534008	5.2 (47)	0.9 (451)	4.3 (10)	0.633	4.238	2.967	2.543	0.286
CHEMBL1315306	5.2 (47)	1.1 (518)	4.1 (11)	0.637	2.004	2.487	2.531	0.258
CHEMBL1493497	5.2 (47)	2 (943)	3.2 (20)	0.547	2.106	1.264	1.702	0.11
CHEMBL1365323	5.1 (46)	2 (971)	3.1 (21)	0.555	2.01	0.867	1.301	0.083

[1] The number of compounds retrieved by the query is reported in round brackets. [2] The number of inactives per active compound is reported in round brackets.

Table S6. Best-three performing queries identified for each cluster in the different datasets of ligands. Only clusters including at least three active compounds were considered for the analyses. The majority of the clusters include ligands able to discriminate actives from inactives with satisfactory screening performances. This is especially true for the clusters generated from the datasets including the most potent Tau aggregation inhibitors, and ligands with a reported Potency value above 10 μM or 20 μM.

Dataset	*Cluster ID*	*Best QUERY*	*AUC*	*EF1*	*EF5*	*EF10*	*BED-ROC*	*Number ofAc-tives Above Thresh.*	*Number ofInac-tives Above Thresh.*	*Number of Com-pounds Above Thresh.*	*Number ofAc-tives Per Cluster*	*Num-ber of Inac-tives Per Cluster*	*Number ofCom-pounds Per Cluster*
A I_ 1μM	2	CHEMBL155868 3	0.679	2.92	2.4	2.512	0.205	99	1443	1542	905	47186	48091
A I_ 1μM	2	CHEMBL144202 3	0.686	5.929	4.68	2.46	0.327	97	1245	1342	905	47186	48091
A I_ 1μM	2	CHEMBL154491 0	0.552	3.971	1.85	1.749	0.158	97	1540	1637	905	47186	48091
A I_ 5μM	2	CHEMBL155868 3	0.69	3.731	2.4	2.42	0.255	99	1009	1108	905	40280	41185
A I_ 5μM	2	CHEMBL144202 3	0.71	6.006	4.7	2.86	0.419	97	874	971	905	40280	41185
A I_ 5μM	2	CHEMBL154491 0	0.554	4.069	1.83	1.744	0.192	97	1087	1184	905	40280	41185
A I_ 10μM	475	CHEMBL140252 9	0.722	3.25	3.25	1.625	0.789	4	9	13	4	9	13
A I_ 10μM	475	CHEMBL152577 6	0.611	3.25	3.25	1.625	0.789	4	9	13	4	9	13
A I_ 10μM	475	CHEMBL147762 8	0.75	3	3	1.5	0.842	4	8	12	4	9	13
A I_ 10μM	1621	CHEMBL154706 4	0.611	3	3	3	0.894	3	6	9	3	10	13
A I_ 10μM	1621	CHEMBL320754 7	0.433	4.333	4.33	2.167	0.793	3	10	13	3	10	13
A I_ 10μM	1621	CHEMBL144844 7	0.367	4.333	4.33	2.167	0.793	3	10	13	3	10	13

A I_ 10µM	2222	CHEMBL1431458	0.842	7.333	3.67	2.444	0.681	3	19	22	3	19	22
A I_ 10µM	2222	CHEMBL1394401	0.789	7.333	3.67	2.444	0.656	3	19	22	3	19	22
A I_ 10µM	2222	CHEMBL3211538	0.842	7.333	7.33	4.889	0.896	3	19	22	3	19	22
A I_ 10µM	2283	CHEMBL1993711	1	2	2	2	1	3	3	6	3	3	6
A I_ 10µM	2283	CHEMBL3189782	0.889	2	2	2	0.999	3	3	6	3	3	6
A I_ 10µM	2283	CHEMBL1976414	0.667	1.667	1.67	1.667	1	3	2	5	3	3	6
A I_ 10µM	2315	CHEMBL1553704	0.722	3.25	3.25	1.625	0.796	4	9	13	4	9	13
A I_ 10µM	2315	CHEMBL1436319	0.722	3.25	3.25	3.25	0.956	4	9	13	4	9	13
A I_ 10µM	2315	CHEMBL1512167	0.722	3.25	3.25	3.25	0.956	4	9	13	4	9	13
A I_ 10µM	2469	CHEMBL1427405	0.706	5.25	2.63	1.75	0.636	4	17	21	4	18	22
A I_ 10µM	2469	CHEMBL1477020	0.792	5.5	5.5	3.667	0.867	4	18	22	4	18	22
A I_ 10µM	2469	CHEMBL1612259	0.618	5.25	2.63	1.75	0.642	4	17	21	4	18	22
A I_ 10µM	2701	CHEMBL1564465	1	1.667	1.67	1.667	1	3	2	5	3	3	6
A I_ 10µM	2701	CHEMBL1412612	1	1.333	1.33	1.333	1	3	1	4	3	3	6
A I_ 10µM	2701	CHEMBL1572149	1	1.333	1.33	1.333	1	3	1	4	3	3	6
A I_ 10µM	2702	CHEMBL1451527	0.86	3	3	3	0.986	5	10	15	6	17	23
A I_ 10µM	2702	CHEMBL1328691	0.891	3.2	3.2	3.2	0.941	5	11	16	6	17	23

A I_ 10μM	2702	CHEMBL1592536	0.833	3.4	3.4	3.4	0.994	5	12	17	6	17	23
A I_ 10μM	2703	CHEMBL1487824	0.8	6	6	3	0.72	3	15	18	3	20	23
A I_ 10μM	2703	CHEMBL1544910	0.843	6.667	6.67	3.333	0.755	3	17	20	3	20	23
A I_ 10μM	2703	CHEMBL1476787	0.846	5.333	5.33	2.667	0.748	3	13	16	3	20	23
A I_ 10μM	2707	CHEMBL1312608	0.72	3	3	3	0.932	5	10	15	5	11	16
A I_ 10μM	2707	CHEMBL1514501	0.72	3	3	3	0.932	5	10	15	5	11	16
A I_ 10μM	2707	CHEMBL1487884	0.727	3.2	3.2	3.2	0.92	5	11	16	5	11	16
A I_ 10μM	3116	CHEMBL428130	0.812	4	4	2	0.778	4	12	16	4	12	16
A I_ 10μM	3116	CHEMBL259807	0.75	4	4	4	0.924	4	12	16	4	12	16
A I_ 10μM	3116	CHEMBL259072	0.833	4	4	2	0.779	4	12	16	4	12	16
A I_ 10μM	3621	CHEMBL1473734	0.63	4	4	2	0.817	3	9	12	3	9	12
A I_ 10μM	3621	CHEMBL1475243	0.63	4	4	2	0.817	3	9	12	3	9	12
A I_ 10μM	3621	CHEMBL1363748	0.63	4	4	2	0.818	3	9	12	3	9	12
A I_ 10μM	3639	CHEMBL1346848	0.833	13	6.5	3.25	0.55	3	36	39	3	39	42
A I_ 10μM	3639	CHEMBL1450911	0.59	14	4.67	2.8	0.499	3	39	42	3	39	42
A I_ 10μM	3639	CHEMBL1455970	0.518	13.67	4.56	2.733	0.502	3	38	41	3	39	42
A I_ 10μM	4748	CHEMBL1514588	0.867	4.75	4.75	4.75	0.895	4	15	19	4	15	19

A I_ 10μM	4748	CHEMBL1518645	0.583	4.75	4.75	4.75	0.891	4	15	19	4	15	19
A I_ 10μM	4748	CHEMBL1473865	0.45	4.75	4.75	2.375	0.661	4	15	19	4	15	19
A I_ 10μM	4860	CHEMBL1349094	0.852	8.5	5.67	2.833	0.651	6	45	51	6	49	55
A I_ 10μM	4860	CHEMBL1512435	0.846	7.667	2.56	4.6	0.574	6	40	46	6	49	55
A I_ 10μM	4860	CHEMBL1590714	0.781	9	6	3	0.592	6	48	54	6	49	55
A I_ 10μM	5027	CHEMBL1590162	0.952	5.667	5.67	5.667	0.939	3	14	17	3	14	17
A I_ 10μM	5027	CHEMBL1551034	0.929	5.667	5.67	5.667	0.934	3	14	17	3	14	17
A I_ 10μM	5027	CHEMBL1358911	0.952	5.667	5.67	2.833	0.801	3	14	17	3	14	17
A I_ 10μM	5182	CHEMBL1410048	0.792	3.667	3.67	3.667	0.978	3	8	11	3	16	19
A I_ 10μM	5182	CHEMBL1583382	0.697	4.667	4.67	2.333	0.815	3	11	14	3	16	19
A I_ 10μM	5182	CHEMBL1474570	0.333	4	4	2	0.817	3	9	12	3	16	19
A I_ 10μM	5206	CHEMBL1598324	0.667	4	4	2	0.818	3	9	12	3	9	12
A I_ 10μM	5206	CHEMBL1367240	0.815	4	4	2	0.846	3	9	12	3	9	12
A I_ 10μM	5206	CHEMBL1394715	0.905	3.333	3.33	3.333	0.984	3	7	10	3	9	12
A I_ 10μM	5713	CHEMBL1417332	0.853	8.25	8.25	4.125	0.775	4	29	33	4	29	33
A I_ 10μM	5713	CHEMBL1415363	0.664	8.25	4.13	4.125	0.58	4	29	33	4	29	33
A I_ 10μM	5713	CHEMBL1434030	0.75	8.25	8.25	4.125	0.771	4	29	33	4	29	33

A I_ 10μM	6055	CHEMBL1512499	0.958	5.5	5.5	5.5	0.963	4	18	22	4	18	22
A I_ 10μM	6055	CHEMBL1554077	0.917	5.5	5.5	5.5	0.96	4	18	22	4	18	22
A I_ 10μM	6055	CHEMBL1591472	0.75	5.5	2.75	1.833	0.615	4	18	22	4	18	22
A I_ 10μM	6163	CHEMBL1481221	0.556	4	4	2	0.817	3	9	12	3	9	12
A I_ 10μM	6163	CHEMBL1366547	0.852	4	4	2	0.823	3	9	12	3	9	12
A I_ 10μM	6163	CHEMBL1554378	0.926	4	4	2	0.851	3	9	12	3	9	12
A I_ 10μM	6178	CHEMBL1590802	0.75	3.5	3.5	1.75	0.809	4	10	14	4	10	14
A I_ 10μM	6178	CHEMBL1363451	0.75	3.5	3.5	3.5	0.946	4	10	14	4	10	14
A I_ 10μM	6178	CHEMBL1435455	0.775	3.5	3.5	1.75	0.776	4	10	14	4	10	14
A I_ 10μM	6179	CHEMBL1331766	0.932	3.75	3.75	3.75	0.95	4	11	15	4	11	15
A I_ 10μM	6179	CHEMBL1589767	0.886	3.75	3.75	3.75	0.986	4	11	15	4	11	15
A I_ 10μM	6179	CHEMBL1364306	0.864	3.75	3.75	3.75	0.986	4	11	15	4	11	15
A I_ 10μM	6184	CHEMBL1528499	1	3.333	3.33	3.333	1	3	7	10	3	7	10
A I_ 10μM	6184	CHEMBL1489661	0.905	3.333	3.33	3.333	0.885	3	7	10	3	7	10
A I_ 10μM	6184	CHEMBL1597278	0.952	3.333	3.33	3.333	0.986	3	7	10	3	7	10
A I_ 10μM	6190	CHEMBL1395139	0.955	3.75	3.75	3.75	0.952	4	11	15	4	11	15
A I_ 10μM	6190	CHEMBL1475385	0.955	3.75	3.75	3.75	0.987	4	11	15	4	11	15

A I_ 10μM	6190	CHEMBL1475 098	0.977	3.75	3.75	3.75	0.99	4	11	15	4	11	15
A I_ 10μM	6213	CHEMBL1472 867	0.872	2.368	2.37	2.368	0.944	19	26	45	19	26	45
A I_ 10μM	6213	CHEMBL1364 914	0.876	2.211	2.21	2.211	0.991	19	23	42	19	26	45
A I_ 10μM	6213	CHEMBL1435 594	0.885	2.158	2.16	2.158	0.992	19	22	41	19	26	45
A I_ 10μM	6514	CHEMBL1610 332	1	6	6	6	1	3	15	18	3	15	18
A I_ 10μM	6514	CHEMBL1606 319	0.889	6	6	6	0.925	3	15	18	3	15	18
A I_ 10μM	6514	CHEMBL1546 678	1	6	6	6	1	3	15	18	3	15	18
A I_ 10μM	7064	CHEMBL1376 988	0.596	7.333	3.67	2.444	0.639	3	19	22	3	19	22
A I_ 10μM	7064	CHEMBL3198 135	0.889	7	3.5	4.667	0.749	3	18	21	3	19	22
A I_ 10μM	7064	CHEMBL1968 356	0.788	4.667	4.67	2.333	0.815	3	11	14	3	19	22
A I_ 10μM	7118	CHEMBL1405 215	0.75	3.25	3.25	3.25	0.956	4	9	13	4	14	18
A I_ 10μM	7118	CHEMBL1368 354	0.806	3.25	3.25	3.25	0.956	4	9	13	4	14	18
A I_ 10μM	7118	CHEMBL1481 415	0.812	3	3	3	0.966	4	8	12	4	14	18
A I_ 10μM	7120	CHEMBL1607 810	0.958	2.125	2.13	2.125	1	8	9	17	8	9	17
A I_ 10μM	7120	CHEMBL1451 292	0.986	2.125	2.13	2.125	1	8	9	17	8	9	17
A I_ 10μM	7120	CHEMBL1577 023	0.875	2.125	2.13	2.125	0.993	8	9	17	8	9	17
A I_ 10μM	7145	CHEMBL1354 702	0.778	3.5	3.5	2.333	0.89	6	15	21	6	15	21

A I_ 10μM	7145	CHEMBL1554186	0.7	3.5	3.5	3.5	0.981	6	15	21	6	15	21
A I_ 10μM	7145	CHEMBL1354557	0.656	3.5	3.5	2.333	0.87	6	15	21	6	15	21
A I_ 10μM	7171	CHEMBL1567897	0.769	1.947	1.95	1.461	0.808	19	18	37	19	20	39
A I_ 10μM	7171	CHEMBL1395944	0.752	1.737	0.87	0.868	0.616	19	14	33	19	20	39
A I_ 10μM	7171	CHEMBL1449627	0.681	1.947	0.97	0.974	0.639	19	18	37	19	20	39
A I_ 10μM	7246	CHEMBL1524383	0.523	4.667	4.67	3.111	0.772	6	22	28	6	22	28
A I_ 10μM	7246	CHEMBL1421060	0.5	4.667	4.67	3.111	0.773	6	22	28	6	22	28
A I_ 10μM	7246	CHEMBL1530146	0.71	5	5	3.333	0.814	5	20	25	6	22	28
A I_ 10μM	7251	CHEMBL1483703	0.773	2.636	2.64	1.758	0.809	11	18	29	11	20	31
A I_ 10μM	7251	CHEMBL1373657	0.732	2.636	2.64	1.758	0.864	11	18	29	11	20	31
A I_ 10μM	7251	CHEMBL1572603	0.732	2.818	2.82	2.818	0.949	11	20	31	11	20	31
A I_ 10μM	7287	CHEMBL1490651	0.979	2.333	2.33	2.333	1	6	8	14	6	8	14
A I_ 10μM	7287	CHEMBL1553703	0.917	2.333	2.33	2.333	0.999	6	8	14	6	8	14
A I_ 10μM	7287	CHEMBL1600901	0.917	2.333	2.33	2.333	0.999	6	8	14	6	8	14
A I_ 10μM	7461	CHEMBL1358532	0.667	4	4	2	0.846	3	9	12	3	9	12
A I_ 10μM	7461	CHEMBL1436190	0.667	4	4	2	0.822	3	9	12	3	9	12
A I_ 10μM	7461	CHEMBL1593148	0.556	4	4	2	0.817	3	9	12	3	9	12

A I_ 10μM	7469	CHEMBL1608606	0.906	4.4	4.4	4.4	0.951	5	17	22	5	17	22
A I_ 10μM	7469	CHEMBL1475016	0.882	4.4	4.4	2.933	0.893	5	17	22	5	17	22
A I_ 10μM	7469	CHEMBL1611467	0.859	4.4	4.4	4.4	0.984	5	17	22	5	17	22
A I_ 10μM	7608	CHEMBL1579296	0.636	2.571	2.57	2.571	0.965	7	11	18	7	11	18
A I_ 10μM	7608	CHEMBL1482801	0.688	2.571	2.57	2.571	0.967	7	11	18	7	11	18
A I_ 10μM	7608	CHEMBL1613377	0.61	2.571	2.57	2.571	0.965	7	11	18	7	11	18
A I_ 10μM	7760	CHEMBL1993796	1	10.8	10.8	9	1	5	49	54	5	55	60
A I_ 10μM	7760	CHEMBL3197583	1	10.8	10.8	9	1	5	49	54	5	55	60
A I_ 10μM	7760	CHEMBL3192848	1	9.8	9.8	9.8	1	5	44	49	5	55	60
A I_ 20μM	732	CHEMBL1554378	0.714	2.75	2.75	1.375	0.864	4	7	11	4	7	11
A I_ 20μM	732	CHEMBL1561032	0.714	2.75	2.75	2.75	0.978	4	7	11	4	7	11
A I_ 20μM	732	CHEMBL1366547	0.786	2.75	2.75	1.375	0.843	4	7	11	4	7	11
A I_ 20μM	746	CHEMBL1363451	0.714	2.75	2.75	2.75	0.974	4	7	11	4	7	11
A I_ 20μM	746	CHEMBL1590802	0.75	2.75	2.75	1.375	0.864	4	7	11	4	7	11
A I_ 20μM	746	CHEMBL1435455	0.75	2.75	2.75	1.375	0.842	4	7	11	4	7	11
A I_ 20μM	1009	CHEMBL1554186	0.722	2	2	2	0.999	6	6	12	6	6	12
A I_ 20μM	1009	CHEMBL1354702	0.75	2	2	2	0.994	6	6	12	6	6	12

A I_ 20µM	1009	CHEMBL1335666	0.806	2	2	2	0.994	6	6	12	6	6	12
A I_ 20µM	1054	CHEMBL1333405	0.524	3.333	3.33	3.333	0.867	3	7	10	3	7	10
A I_ 20µM	1054	CHEMBL1514506	0.762	3.333	3.33	3.333	0.883	3	7	10	3	7	10
A I_ 20µM	1054	CHEMBL1347146	0.667	2.333	2.33	2.333	0.997	3	4	7	3	7	10
A I_ 20µM	1069	CHEMBL1318268	0.8	2	2	2	0.998	5	5	10	5	7	12
A I_ 20µM	1069	CHEMBL1333313	0.829	2.4	2.4	2.4	0.994	5	7	12	5	7	12
A I_ 20µM	1069	CHEMBL1338662	0.829	2.4	2.4	2.4	0.994	5	7	12	5	7	12
A I_ 20µM	1077	CHEMBL1524383	0.833	2.125	2.13	2.125	0.978	8	9	17	8	9	17
A I_ 20µM	1077	CHEMBL1421060	0.778	2.125	2.13	2.125	0.997	8	9	17	8	9	17
A I_ 20µM	1077	CHEMBL1413229	0.75	2	2	2	0.995	8	8	16	8	9	17
A I_ 20µM	1190	CHEMBL1441761	0.667	3.333	3.33	3.333	0.883	3	7	10	3	7	10
A I_ 20µM	1190	CHEMBL1472767	0.667	3.333	3.33	3.333	0.869	3	7	10	3	7	10
A I_ 20µM	1190	CHEMBL1466774	0.524	3.333	3.33	3.333	0.867	3	7	10	3	7	10
A I_ 20µM	1199	CHEMBL1530889	0.953	2	2	2	0.999	8	8	16	8	8	16
A I_ 20µM	1199	CHEMBL1577023	0.938	2	2	2	0.995	8	8	16	8	8	16
A I_ 20µM	1199	CHEMBL1607810	0.953	2	2	2	1	8	8	16	8	8	16
A I_ 20µM	1208	CHEMBL1475098	0.964	2.75	2.75	2.75	0.997	4	7	11	4	7	11

A I_ 20μM	1208	CHEMBL1395139	0.929	2.75	2.75	2.75	0.978	4	7	11	4	7	11
A I_ 20μM	1208	CHEMBL1475385	0.929	2.75	2.75	2.75	0.997	4	7	11	4	7	11
A I_ 20μM	1448	CHEMBL1489732	0.767	7.667	3.83	2.556	0.673	3	20	23	3	21	24
A I_ 20μM	1448	CHEMBL1529255	0.487	5.333	5.33	2.667	0.731	3	13	16	3	21	24
A I_ 20μM	1448	CHEMBL1306892	0.667	8	4	2.667	0.62	3	21	24	3	21	24
A I_ 20μM	1696	CHEMBL3192848	1	5.2	5.2	5.2	1	5	21	26	5	25	30
A I_ 20μM	1696	CHEMBL3197583	1	5.6	5.6	5.6	1	5	23	28	5	25	30
A I_ 20μM	1696	CHEMBL1993796	1	5.6	5.6	5.6	1	5	23	28	5	25	30
A I_ 20μM	1768	CHEMBL1613377	0.732	2.143	2.14	2.143	0.983	7	8	15	7	8	15
A I_ 20μM	1768	CHEMBL1482801	0.768	2.143	2.14	2.143	0.985	7	8	15	7	8	15
A I_ 20μM	1768	CHEMBL1373186	0.696	2.143	2.14	2.143	0.944	7	8	15	7	8	15
A I_ 20μM	1862	CHEMBL1417332	0.867	4.75	4.75	4.75	0.901	4	15	19	4	15	19
A I_ 20μM	1862	CHEMBL1434030	0.783	4.75	4.75	4.75	0.892	4	15	19	4	15	19
A I_ 20μM	1862	CHEMBL1415363	0.683	4.75	4.75	2.375	0.741	4	15	19	4	15	19
A I_ 20μM	2127	CHEMBL546649	0.667	2.333	2.33	2.333	0.946	3	4	7	3	4	7
A I_ 20μM	2127	CHEMBL1340539	0.583	2.333	2.33	2.333	0.946	3	4	7	3	4	7
A I_ 20μM	2127	CHEMBL1558403	0.333	2.333	2.33	2.333	0.943	3	4	7	3	4	7

A I_ 20μM	2623	CHEMBL1590162	1	5	5	5	1	4	16	20	4	17	21
A I_ 20μM	2623	CHEMBL1358911	1	5	5	5	1	4	16	20	4	17	21
A I_ 20μM	2623	CHEMBL1551034	0.953	5	5	5	0.917	4	16	20	4	17	21
A I_ 20μM	3346	CHEMBL3211538	0.846	5.333	5.33	5.333	0.94	3	13	16	3	13	16
A I_ 20μM	3346	CHEMBL1394401	0.846	5.333	5.33	2.667	0.791	3	13	16	3	13	16
A I_ 20μM	3346	CHEMBL1431458	0.872	5.333	5.33	2.667	0.749	3	13	16	3	13	16
A I_ 20μM	3713	CHEMBL1328691	0.8	2	2	2	0.984	5	5	10	5	6	11
A I_ 20μM	3713	CHEMBL1451527	0.84	2	2	2	0.998	5	5	10	5	6	11
A I_ 20μM	3713	CHEMBL1592536	0.867	2.2	2.2	2.2	0.999	5	6	11	5	6	11
A I_ 20μM	3714	CHEMBL1487824	0.607	2.75	2.75	1.375	0.86	4	7	11	5	11	16
A I_ 20μM	3714	CHEMBL1982450	0.792	2.5	2.5	2.5	0.982	4	6	10	5	11	16
A I_ 20μM	3714	CHEMBL1476787	0.567	2.2	2.2	1.1	0.842	5	6	11	5	11	16
A I_ 20μM	3716	CHEMBL1312608	0.88	2	2	2	0.984	5	5	10	5	6	11
A I_ 20μM	3716	CHEMBL1514501	0.733	2.2	2.2	2.2	0.974	5	6	11	5	6	11
A I_ 20μM	3716	CHEMBL1487884	0.8	2.2	2.2	2.2	0.978	5	6	11	5	6	11
A I_ 20μM	3909	CHEMBL1553704	0.875	2.5	2.5	2.5	0.998	4	6	10	4	6	10
A I_ 20μM	3909	CHEMBL1512167	0.792	2.5	2.5	2.5	0.984	4	6	10	4	6	10

A I_ 20μM	3909	CHEMBL1436 319	0.792	2.5	2.5	2.5	0.984	4	6	10	4	6	10
A I_ 20μM	4750	CHEMBL1387 425	0.833	7	3.5	4.667	0.749	3	18	21	3	18	21
A I_ 20μM	4750	CHEMBL1455 551	0.979	6.333	6.33	6.333	0.946	3	16	19	3	18	21
A I_ 20μM	4750	CHEMBL1490 176	0.778	7	3.5	2.333	0.666	3	18	21	3	18	21
A I_ 20μM	4771	CHEMBL1507 672	0.978	6	6	6	0.949	3	15	18	3	20	23
A I_ 20μM	4771	CHEMBL1447 971	0.867	7.667	3.83	2.556	0.639	3	20	23	3	20	23
A I_ 20μM	4771	CHEMBL1421 876	0.98	6.667	6.67	6.667	0.943	3	17	20	3	20	23
A I_ 20μM	4970	CHEMBL1346 848	0.897	5.333	5.33	2.667	0.792	3	13	16	3	13	16
A I_ 20μM	4970	CHEMBL1450 911	0.513	5.333	5.33	2.667	0.731	3	13	16	3	13	16
A I_ 20μM	4970	CHEMBL1455 970	0.385	5.333	5.33	2.667	0.731	3	13	16	3	13	16
A I_ 20μM	4988	CHEMBL1552 151	0.917	2.333	2.33	2.333	0.997	3	4	7	3	4	7
A I_ 20μM	4988	CHEMBL1437 180	0.75	2.333	2.33	2.333	0.997	3	4	7	3	4	7
A I_ 20μM	4988	CHEMBL1400 574	0.917	2.333	2.33	2.333	0.997	3	4	7	3	4	7
A I_ 20μM	4990	CHEMBL1473 734	0.667	3	3	3	0.893	3	6	9	3	6	9
A I_ 20μM	4990	CHEMBL1475 243	0.667	3	3	3	0.893	3	6	9	3	6	9
A I_ 20μM	4990	CHEMBL1363 748	0.556	3	3	3	0.893	3	6	9	3	6	9
A I_ 20μM	5223	CHEMBL1583 382	0.714	3.333	3.33	3.333	0.883	3	7	10	3	12	15

A I_ 20μM	5223	CHEMBL1474570	0.333	3	3	3	0.893	3	6	9	3	12	15
A I_ 20μM	5223	CHEMBL1410048	0.667	2.667	2.67	2.667	0.994	3	5	8	3	12	15
A I_ 20μM	5235	CHEMBL1394715	0.933	2.667	2.67	2.667	0.994	3	5	8	3	6	9
A I_ 20μM	5235	CHEMBL1367240	0.889	3	3	3	0.99	3	6	9	3	6	9
A I_ 20μM	5235	CHEMBL1598324	0.556	3	3	3	0.893	3	6	9	3	6	9
A I_ 20μM	5406	CHEMBL1525776	0.712	4.25	4.25	2.125	0.705	4	13	17	4	13	17
A I_ 20μM	5406	CHEMBL1477628	0.731	4.25	4.25	2.125	0.719	4	13	17	4	13	17
A I_ 20μM	5406	CHEMBL1402529	0.808	4.25	4.25	2.125	0.706	4	13	17	4	13	17
A I_ 20μM	5410	CHEMBL1411308	0.481	4	4	2	0.817	3	9	12	3	9	12
A I_ 20μM	5410	CHEMBL1560910	0.407	4	4	2	0.817	3	9	12	3	9	12
A I_ 20μM	5410	CHEMBL1348909	0.556	4	4	2	0.818	3	9	12	3	9	12
A I_ 20μM	5418	CHEMBL259807	0.8	2.25	2.25	2.25	0.989	4	5	9	4	5	9
A I_ 20μM	5418	CHEMBL259072	0.9	2.25	2.25	2.25	0.99	4	5	9	4	5	9
A I_ 20μM	5418	CHEMBL428130	0.9	2.25	2.25	2.25	0.99	4	5	9	4	5	9

Table S7. Results of the ECFP4*fp*-based similarity screenings obtained for the best performing set of queries *per* combination.

Number of queries per combination	*Queries*	*AUC*	*EF (1%)*	*EF (5%)*	*EF (10%)*	*BEDROC*
1	CHEMBL1558683	0.676	3	2.429	2.036	0.185
2	CHEMBL1544910-CHEMBL1591502	0.566	3.541	1.703	1.399	0.155
3	CHEMBL1544910-CHEMBL1431458-CHEMBL1591502	0.568	3.748	1.721	1.339	0.14
4	CHEMBL1544910-CHEMBL1431458-CHEMBL1572407-CHEMBL3194159	0.561	4.64	1.519	1.35	0.135
5	CHEMBL1544910-CHEMBL1431458-CHEMBL3194159-CHEMBL1572149-CHEMBL1328302	0.562	4.102	1.968	1.438	0.143
6	CHEMBL1558683-CHEMBL1544910-CHEMBL1431458-CHEMBL3194159-CHEMBL1572149-CHEMBL1328302	0.586	4.577	2.212	1.677	0.155
7	CHEMBL1558683-CHEMBL1544910-CHEMBL1431458-CHEMBL1572407- CHEMBL1572149-CHEMBL1328302-CHEMBL3194482	0.596	5.645	1.938	1.504	0.156
8	CHEMBL1558683-CHEMBL1544910-CHEMBL1442023-CHEMBL1354557- CHEMBL1431458-CHEMBL1564465-CHEMBL1328302-CHEMBL3194482	0.62	6.397	3.241	2.026	0.204
9	CHEMBL1558683-CHEMBL1544910-CHEMBL1442023-CHEMBL1476765- CHEMBL1431458-CHEMBL1572407-CHEMBL1564465-CHEMBL1328302- CHEMBL3194482	0.634	7.461	3.058	2.15	0.211
10	CHEMBL1558683-CHEMBL1544910-CHEMBL1442023-CHEMBL1476765- CHEMBL1355273-CHEMBL1431458-CHEMBL1572407-CHEMBL1564465- CHEMBL1328302-CHEMBL3194482	0.638	6.089	3.628	2.303	0.227
11	CHEMBL1558683-CHEMBL1544910-CHEMBL1442023-CHEMBL1476765- CHEMBL1355273-CHEMBL1555206-CHEMBL1431458-CHEMBL1572407- CHEMBL1564465-CHEMBL1328302-CHEMBL3194482	0.639	6.422	3.606	2.289	0.226

12	CHEMBL1558683-CHEMBL1544910-CHEMBL1442023-CHEMBL1476765-CHEMBL1355273-CHEMBL1555206-CHEMBL1354557-CHEMBL1431458- CHEMBL1572407-CHEMBL1564465-CHEMBL1328302-CHEMBL3194482	0.643	6.908	3.599	2.402	0.234
13	CHEMBL1558683-CHEMBL1544910-CHEMBL1442023-CHEMBL1476765- CHEMBL1355273-CHEMBL1555206-CHEMBL1354557-CHEMBL1431458- CHEMBL1572407-CHEMBL1564465-CHEMBL1328302-CHEMBL1535640-CHEMBL3194482	0.65	7.038	3.733	2.512	0.24
14	CHEMBL1558683-CHEMBL1544910-CHEMBL1442023-CHEMBL1476765-CHEMBL1355273-CHEMBL1555206-CHEMBL1354557-CHEMBL1431458- CHEMBL1405215-CHEMBL1572407-CHEMBL1564465-CHEMBL1328302- CHEMBL1535640-CHEMBL3194482	0.654	7.335	3.799	2.513	0.242
15	CHEMBL1558683-CHEMBL1544910-CHEMBL1442023-CHEMBL1476765-CHEMBL1355273-CHEMBL1555206-CHEMBL1354557-CHEMBL1431458- CHEMBL1405215-CHEMBL1607810-CHEMBL1572407-CHEMBL1564465- CHEMBL1328302-CHEMBL1535640-CHEMBL3194482	0.652	8.255	3.913	2.538	0.249
16	CHEMBL1558683-CHEMBL1544910-CHEMBL1442023-CHEMBL1476765-CHEMBL1355273-CHEMBL1555206-CHEMBL1354557-CHEMBL1431458- CHEMBL1405215-CHEMBL1607810-CHEMBL1572407-CHEMBL1592536- CHEMBL1564465-CHEMBL1328302-CHEMBL1535640-CHEMBL3194482	0.656	8.406	3.973	2.678	0.254
17	CHEMBL1558683-CHEMBL1544910-CHEMBL1442023-CHEMBL1476765- CHEMBL1355273-CHEMBL1555206-CHEMBL1354557-CHEMBL1431458- CHEMBL1405215-CHEMBL1607810-CHEMBL1572407-CHEMBL1592536-CHEMBL3194159-CHEMBL1564465-CHEMBL1515009-CHEMBL1328302- CHEMBL3194482	0.655	7.919	3.958	2.721	0.256

18	CHEMBL1558683-CHEMBL1544910-CHEMBL1442023-CHEMBL1476765-CHEMBL1355273-CHEMBL1555206-CHEMBL1354557-CHEMBL1431458- CHEMBL1405215-CHEMBL1607810-CHEMBL1572407-CHEMBL1592536- CHEMBL1530889-CHEMBL3194159-CHEMBL1564465-CHEMBL1515009-CHEMBL1328302-CHEMBL3194482	0.656	8.527	4.018	2.692	0.258
19	CHEMBL1558683-CHEMBL1544910-CHEMBL1442023-CHEMBL1476765-CHEMBL1355273-CHEMBL1555206-CHEMBL1354557-CHEMBL1431458- CHEMBL1405215-CHEMBL1607810-CHEMBL1572407-CHEMBL1592536- CHEMBL1530889-CHEMBL1591502-CHEMBL3194159-CHEMBL1564465-CHEMBL1515009-CHEMBL1328302-CHEMBL3194482	0.663	8.749	4.112	2.764	0.259
20	CHEMBL1558683-CHEMBL1544910-CHEMBL1442023-CHEMBL1476765- CHEMBL1355273-CHEMBL1555206-CHEMBL1354557-CHEMBL1431458- CHEMBL1405215-CHEMBL1607810-CHEMBL1572407-CHEMBL1592536-CHEMBL1530889-CHEMBL1591502-CHEMBL3194159-CHEMBL1495007- CHEMBL1564465-CHEMBL1328302-CHEMBL1339146-CHEMBL3194482	0.651	9.305	4.051	2.704	0.261

Table S8. Percentages of Tau aggregation inhibitors that present good drug-like properties according to commonly recommended values, as evaluated with *QikProp* (Schrödinger 2020-1). The total number of active compounds considered in the analyses is 905. The number of inactive compounds is 47246, 40325, 35448, and 16034 at the ≥1μM, ≥5μM ≥10μM and ≥20μM activity thresholds, respectively. The number of ligands with property values within the recommended values is reported in brackets. As shown in the table, the majority of compounds present good drug-like properties (*e.g.*, "*stars*", "*RuleOfFive*" and "*RuleOfThree*"). Moreover, the compounds were also predicted to have good oral-availability (see "*Percentage HumanOralAbsorption*") and to efficiently cross the blood-brain-barrier (BBB) (see "*QPlogBB*" and "*QPPMDCK*"). The fact that good oral adsorption and BBB penetration were predicted for the most active compounds of the series is encouraging for the purposes of this analysis, as such properties are desired for candidate inhibitors of Tau aggregation. Of note, only a small percentage of the activecompounds were predicted to present central nervous system (CNS) activity, as defined by Ajay *et al*. (*J. Med. Chem.* 1999, **42**, 24:4942–4951). Such a result should not be considered an issue because the set of CNS therapeutic classes included in the *QikProp* filters does not include Tau and related disorders.

Descriptor	Actives (≤500nM)	Inactives (≥1μM)	Inactives (≥5μM)	Inactives (≥10μM)	Inactives (≥20μM)
Stars [1]	98.6 (892)	97.7 (46092)	97.6 (39302)	97.5 (34526)	97.4 (15594)
RuleOfFive [2]	97.0 (878)	95.9 (45238)	95.7 (38566)	95.7 (33874)	95.3 (15257)
RuleOfThree [3]	96.4 (872)	95.0 (44825)	94.8 (38171)	94.7 (33520)	94.7 (15167)
RtvFG [4]	98.2 (889)	97.4 (45970)	97.3 (39194)	97.3 (34442)	97.2 (15564)
CNS [5]	12.5 (113)	19.2 (9071)	20 (8045)	20.5 (7261)	22.1 (3541)
QPlogHERG [6]	18.8 (170)	23.5 (11098)	24.8 (9987)	25.4 (8997)	28.1 (4500)
QPlogBB [7]	98.2 (889)	97.5 (45991)	97.3 (39212)	97.3 (34451)	97.3 (15572)
QPPMDCK [8]	60.6 (548)	58.6 (27641)	58.5 (23569)	58.4 (20679)	59.7 (9552)
Metab [9]	96.0 (869)	94.3 (44493)	94.1 (37922)	94.1 (33308)	94 (15049)
QPlogKhsa [10]	98.7 (893)	97.6 (46036)	97.5 (39262)	97.4 (34496)	97.3 (15582)
Percentage Human Oral Absorption [11]	88.5 (801)	84.3 (39756)	83.8 (33746)	83.7 (29636)	84 (13450)

[1] Percentage of ligands with molecular properties not exceeding 5 violations with respect to 95% of known drugs. [2] Percentage of ligands with molecular properties exceeded one violation with respect to the Lipinski's rule of five. (*Adv. Drug Delivery Rev.* **2001**, 46:3-26.). [3] Percentage of ligands with molecular properties exceeding 5 violations with respect to the Jorgensen's rule of three. [4] Percentage of ligands with a number of reactive functional groups outside the range 0 – 2. [5] Percentage of ligands predicted to be active in the CNS (property value higher than 1). [6] Percentage of ligands without concerns with respect to blockade of HERG K^+ channels. [7] Percentage of ligands with predicted brain/blood partition coefficient in the range –3.0-1.2. [8] Percentage of ligands with predicted high (≥500) apparent MDCK cell permeability, which is considered a goodmodel to mimic the BBB. [9] Percentage of ligands that are likely to be metabolized (number of potential metabolic reaction in the range 1-8). [10] Percentage of ligands predicted to bind to human serum albumin (property range –1.5 – 1.5). [11] Percentage of ligands predicted to be orally absorbed in a percentage higher than 80%.

Table S9. Mean (± Standard deviation), and 10th and 90th percentiles evaluated for the molecular descriptors that emerged as different for the actives and inactive compounds.

Descriptor	*Mean ± St.D.(actives)*	*10th-90th (actives)*	*Mean ± St.D.(≥ 1 μM)*	*10th-90th (≥ 1 μM)*	*Mean ± St.D.(≥ 5 μM)*	*10th-90th (≥ 5 μM)*	*Mean ± St.D.(≥ 10 μM)*	*10th-90th (≥ 10 μM)*	*Mean ± St.D.(≥ 20 μM)*	*10th-90th (≥ 20μM)*
Chi0v	16 ± 3.7	12.1–19.8	15.1 ± 3.4	11–19.3	15.1 ± 3.4	10.9–19.3	15.1 ± 3.5	10.9–19.3	15.2 ± 3.6	10.9–19.5
Chi3n	4.5 ± 1.5	3–6	4.2 ± 1.4	2.7–5.9	4.2 ± 1.4	2.7–5.9	4.2 ± 1.4	2.6–5.9	4.3 ± 1.5	2.6–6
kappa2	8 ± 2.5	5.6–10.4	7.4 ± 2	5–9.9	7.4 ± 2.1	5–9.9	7.4 ± 2.1	5–9.9	7.5 ± 2.2	5–10
MQN10	2.7 ± 1.6	1–5	2.4 ± 1.6	1–4	2.4 ± 1.7	0–4	2.4 ± 1.7	0–4	2.4 ± 1.7	0–4
MQN11	0.5 ± 0.8	0–2	0.4 ± 0.7	0–1	0.4 ± 0.7	0–1	0.4 ± 0.7	0–1	0.4 ± 0.7	0–1
MQN13	8.7 ± 3.7	5–13	8 ± 3.3	4–12	8 ± 3.3	4–12	8 ± 3.3	4–12	8.2 ± 3.5	4–12
MQN16	11.4 ± 3.9	8–16	11 ± 4	6–16	11 ± 4.1	6–16	11 ± 4.1	6–16	11 ± 4.3	6–17
MQN27	3.5 ± 2.4	1–6	3 ± 2.1	1–6	3 ± 2.1	1–6	3 ± 2.1	1–6	3 ± 2.2	1–6
MQN28	1.3 ± 1	0–2	1.2 ± 1	0–2	1.2 ± 1	0–2	1.2 ± 1	0–2	1.2 ± 1.1	0–2
MQN30	11.3 ± 3.2	7–15	10.4 ± 3.5	6–15	10.3 ± 3.5	6–15	10.3 ± 3.6	6–15	10.3 ± 3.7	6–15
MQN31	7.4 ± 2.4	5–10	7.1 ± 2.4	4–10	7.1 ± 2.5	4–10	7.1 ± 2.5	4–10	7 ± 2.6	4–10
MQN36	2.6 ± 1	1–4	2.4 ± 1	1–4	2.4 ± 1	1–4	2.4 ± 1	1–4	2.3 ± 1.1	1–4
MQN41	1.7 ± 1.9	0–4	1.5 ± 1.8	0–4	1.5 ± 1.8	0–4	1.5 ± 1.8	0–4	1.5 ± 1.8	0–4
Num Amide Bonds	0.9 ± 0.8	0–2	0.7 ± 0.8	0–2	0.7 ± 0.9	0–2	0.7 ± 0.9	0–2	0.7 ± 0.9	0–2
Num Aromatic Carbocycles	1.8 ± 0.8	1–3	1.6 ± 0.8	1–2	1.6 ± 0.8	1–2	1.6 ± 0.8	1–2	1.5 ± 0.8	1–2
Num Aromatic Heterocycles	1.2 ± 0.8	0–2	1.1 ± 0.9	0–2	1.1 ± 0.9	0–2	1.1 ± 0.9	0–2	1.1 ± 0.9	0–2
Num Aromatic Rings	3 ± 1	2–4	2.6 ± 1	1–4	2.6 ± 1	1–4	2.6 ± 1	1–4	2.6 ± 1	1–4
Num HBA	5.5 ± 2	3–8	5.2 ± 1.8	3–7	5.2 ± 1.9	3–7	5.2 ± 1.9	3–7	5.1 ± 1.9	3–7
Num Hetero Atoms	7.2 ± 2.3	5–10	6.8 ± 2.2	4–10	6.8 ± 2.2	4–10	6.7 ± 2.2	4–9	6.7 ± 2.3	4–9
Num Lipinski HBA	6.2 ± 2.2	4–9	5.8 ± 2	3–8	5.8 ± 2.1	3–8	5.8 ± 2.1	3–8	5.7 ± 2.1	3–8
Num Rings	3.6 ± 1	2–5	3.3 ± 1	2–5	3.3 ± 1.1	2–5	3.3 ± 1.1	2–5	3.3 ± 1.1	2–5
Num Rotatable Bonds	5.4 ± 2.8	2–8	4.7 ± 2.3	2–8	4.7 ± 2.3	2–8	4.7 ± 2.3	2–8	4.8 ± 2.4	2–8

peoe_VSA1	12.8 ± 7.5	4.7–20.1	11.6 ± 6.9	4.6–19.8	11.6 ± 6.9	4.6–19.8	11.6 ± 6.9	4.6–19.9	11.6 ± 7.1	4.6–19.9
peoe_VSA11	5.9 ± 6.4	0–16.5	5.1 ± 6	0–11.6	5.1 ± 6	0–11.6	5 ± 6	0–11.6	4.9 ± 5.9	0–11.6
peoe_VSA12	4.7 ± 5.3	0–11.8	3.8 ± 5	0–11.7	3.8 ± 5.1	0–11.7	3.8 ± 5	0–11.7	4 ± 5.2	0–11.8
peoe_VSA13	4.6 ± 4.7	0–11.6	3.8 ± 4.5	0–11.5	3.7 ± 4.5	0–11.5	3.6 ± 4.5	0–11.5	3.5 ± 4.4	0–11.4
peoe_VSA3	5.6 ± 5.1	0–12.7	4.9 ± 4.8	0–10.1	4.9 ± 4.8	0–10.2	4.9 ± 4.8	0–10.2	5 ± 4.9	0–10.2
peoe_VSA7	43.8 ± 16.3	23.8–63.6	38.5 ± 15.8	18.6–60	38.2 ± 15.9	18.4–59.1	38.1 ± 15.9	18.3–58.6	38.1 ± 16.1	18.2–59.4
SlogP	3.8 ± 1.3	2.1–5.4	3.6 ± 1.3	2–5.1	3.6 ± 1.3	2–5.1	3.6 ± 1.3	2–5.1	3.5 ± 1.3	1.9–5
slogp_VSA1	10.1 ± 6.1	4.7–19.1	8.8 ± 6.1	0–16	8.7 ± 6.1	0–16	8.7 ± 6.1	0–16	8.5 ± 6.1	0–15.8
slogp_VSA11	4.7 ± 5.5	0–11.5	4.1 ± 5.7	0–11.5	4.1 ± 5.7	0–11.5	4 ± 5.7	0–11.5	3.8 ± 5.5	0–11.5
slogp_VSA6	53.9 ± 19.5	28.8–77.5	49.5 ± 18.7	24.3–72.8	49.1 ± 18.9	24.3–72.8	48.9 ± 19	24.3–72.8	48.1 ± 19.4	24.3–72.8
slogp_VSA8	10.1 ± 8.9	0–22.6	8.3 ± 8.4	0–21.8	8 ± 8.3	0–21.7	7.9 ± 8.2	0–21.6	7.6 ± 8.1	0–21.3
smr_VSA1	14.6 ± 8.4	4.8–23.8	13 ± 8.2	4.7–23.5	12.9 ± 8.2	4.7–23.6	12.9 ± 8.2	4.7–23.6	12.9 ± 8.5	4.7–23.6
smr_VSA3	9.9 ± 7	0–19.6	8.7 ± 6.6	0–18.9	8.8 ± 6.7	0–19.1	8.8 ± 6.7	0–19.3	9.2 ± 6.9	0–19.6
smr_VSA9	9.1 ± 8.4	0–22.6	7.7 ± 8.2	0–17.4	7.5 ± 8.2	0–17.3	7.4 ± 8.1	0–17.3	7.1 ± 8	0–17.3
Hall-Kier Alpha[1]	−3 ± 0.9	−4–−1.9	-2.8 ± 0.8	−3.8–−1.8	−2.8 ± 0.8	−3.8–−1.7	−2.7 ± 0.8	−3.8–−1.7	−2.7 ± 0.8	−3.7–−1.6
Num Aliphatic Heterocycles[1]	0.5 ± 0.7	0–1	0.5 ± 0.7	0–1	0.6 ± 0.7	0–1	0.6 ± 0.7	0–1	0.6 ± 0.7	0–2
Num Aliphatic Rings[1]	0.6 ± 0.9	0–2	0.7 ± 0.8	0–2	0.7 ± 0.8	0–2	0.7 ± 0.8	0–2	0.8 ± 0.9	0–2
Num Saturated Heterocycles[1]	0.2 ± 0.5	0–1	0.3 ± 0.5	0–1	0.3 ± 0.5	0–1	0.3 ± 0.5	0–1	0.3 ± 0.6	0–1
smr_VSA2[1]	0.2 ± 1	0–0	0.5 ± 1.8	0–0	0.5 ± 1.8	0–0	0.5 ± 1.8	0–0	0.5 ± 2	0–0
Chi3[2]	5.2 ± 1.6	3.4–7	4.9 ± 1.5	3.1–6.8	4.9 ± 1.6	3.1–6.9	4.9 ± 1.6	3.1–6.9	5 ± 1.6	3.1–7
MQN24[3]	0.1 ± 0.3	0–0	0.1 ± 0.4	0–1	0.1 ± 0.4	0–1	0.1 ± 0.4	0–1	0.1 ± 0.3	0–0
Num Saturated Rings[3]	0.3 ± 0.7	0–1	0.4 ± 0.6	0–1	0.4 ± 0.6	0–1	0.4 ± 0.6	0–1	0.4 ± 0.7	0–1

Note: [1] Molecular descriptors with values that resulted to be lower for the active compounds, with respect to inactives at the 1 μM, 5 μM, 10 μM and 20 μM activity thresholds. [2] Molecular descriptors with values that resulted to be higher for the active compounds, with respect to inactives at the 1 μM, 5 μM,

and 10 μM activity thresholds. [3] Molecular descriptors with values that resulted to be lower for the active compounds, with respect to inactives at the 5 μM, 10 μM and 20 μM activity thresholds.

Table S10. Percentage of compounds with molecular descriptors within the 10th and 90th percentile thresholds of the active compounds.

Descriptor	*Actives (≤500nM)*	*Inactives (≥1nM)*	*Inactives (≥5nM)*	*Inactives (≥10nM)*	*Inactives (≥20nM)*
**ALL**	**48.62**	**31.52**	**30.26**	**29.59**	**27.91**
Chi0v	89,9	81,2	80,7	80,6	80,6
Chi3n	89,9	84,0	83,5	83,3	83,4
kappa2	89,9	83,0	82,6	82,5	82,5
MQN10	95,0	90,2	89,9	89,8	89,5
MQN11	100,0	100,0	100,0	100,0	100,0
MQN13	91,6	87,4	87,5	87,5	87,8
MQN16	90,2	82,8	82,1	81,8	71,1
MQN27	94,5	91,3	91,1	91,0	90,9
MQN28	100,0	100,0	100,0	100,0	100,0
MQN30	93,5	87,5	86,9	86,6	85,5
MQN31	90,8	86,8	86,2	85,9	85,0
MQN36	99,0	97,9	97,8	97,7	97,0
MQN41	100,0	100,0	100,0	100,0	100,0
Num Amide Bonds	100,0	100,0	100,0	100,0	100,0
Num Aromatic Carbocycles	94,3	92,9	92,5	92,2	90,6
Num Aromatic Heterocycles	100,0	100,0	100,0	100,0	100,0
Num Aromatic Rings	92,2	89,8	89,2	88,8	87,2
Num HBA	96,8	94,7	94,6	94,5	93,7
Num Hetero Atoms	91,3	85,9	85,5	85,4	84,8
Num Lipinski HBA	92,4	87,9	87,6	87,5	86,7
Num Rings	98,5	97,6	97,4	97,4	96,9
Num Rotatable Bonds	97,2	94,6	94,5	94,4	94,3
peoe_VSA1	93,0	89,7	89,7	89,7	89,5
peoe_VSA11	100,0	100,0	100,0	100,0	100,0
peoe_VSA12	100,0	100,0	100,0	100,0	100,0
peoe_VSA13	100,0	100,0	100,0	100,0	100,0
peoe_VSA3	100,0	100,0	100,0	100,0	100,0
peoe_VSA7	89,9	85,1	84,5	84,3	83,6
SlogP	89,9	88,6	88,1	87,9	87,3
slogp_VSA1	90,1	85,0	84,5	84,2	83,4
slogp_VSA11	100,0	100,0	100,0	100,0	100,0
slogp_VSA6	90,1	87,4	86,8	86,4	84,8
slogp_VSA8	100,0	100,0	100,0	100,0	100,0
smr_VSA1	93,1	87,8	87,4	87,3	86,8
smr_VSA3	100,0	100,0	100,0	100,0	100,0
smr_VSA9	100,0	100,0	100,0	100,0	100,0
Hall-Kier Alpha [1]	90,1	86,5	85,8	85,3	83,2
Num Aliphatic Heterocycles [1]	93,3	91,4	91,0	90,8	89,9
Num Aliphatic Rings [1]	96,5	97,1	97,0	96,9	96,5
Num Saturated Heterocycles [1]	98,2	97,3	97,1	97,0	96,7
smr_VSA2 [1]	96,9	92,7	92,6	92,5	92,1

Chi3 [2]	89,9	83,6	83,3	83,3	82,9
MQN24 [3]	93,4	88,1	88,3	88,6	90,1
Num Saturated Rings [3]	95,6	95,5	95,5	95,4	94,7

Notes: [1] Molecular descriptors with values that resulted to be lower for the active compounds, with respect to inactives atthe 1 μM, 5 μM, 10 μM and 20 μM activity thresholds. [2] Molecular descriptors with values that resulted to be higher for the active compounds, with respect to inactives atthe 1 μM, 5 μM, and 10 μM activity thresholds. [3] Molecular descriptors with values that resulted to be lower for the active compounds, with respect to inactives atthe 5 μM, 10 μM and 20 μM activity thresholds.

Table S11. Molecular fragments observed exclusively in the active compounds.

Fragment ID	SMILES
OA1	Cc1nc2c3cn[nH]c3ncn2n1
OA2	OCC1=Nc2c3cnnc-3ncn2[N]1
OA3	OCc1nc2c3cn[nH]c3ncn2n1
OA4	O=CC1=CC[C@@H](CCCO)CO1
OA5	OCCC[C@@H]1[CH]C=CO[CH]1
OA6	OCCC[C@@H]1CC=CO[C@H]1O
OA7	OCCC[C@H]1[CH]OC=CC1
OA8	O=CC1=CC[C@@H](CCCO)[CH]O1
OA9	O=CC1=C[CH][C@@H](CCCO)CO1
OA10	O=CC1=C[CH][C@@H](CCCO)[CH]O1
OA11	OCCC[C@@H]1[CH]C=COC1
OA12	OCCC[C@@H]1CC=COC1
OA13	OCCC[C@@H]1[CH]C=CO[C@H]1O
OA14	c1ncc(NC2CCCCC2)o1
OA15	[CH]1C[C@@H](C2CCCCC2)C=CO1
OA16	O=CC1=C[C@H](C2CCCCC2)C[CH]O1
OA17	Nc1ccc(-c2cn3cccnc3n2)cc1
OA18	CC(=O)Nc1cccc(C(=O)OCc2cc(=O)n3cc(C)sc3n2)c1
OA19	O=CC1=C[CH][CH][CH]O1
OA20	CCCC(=O)N1CCN(c2ccc(C(C)=O)cc2)CC1
OA21	Cc1nc2c3cnn(-c4cccc(F)c4)c3ncn2n1
OA22	c1ccc(CCc2nc3ccc(CNc4ccccc4)cc3o2)cc1
OA23	c1ccc(CNc2ccc(-c3cn4cccnc4n3)cc2)cc1
OA24	C(=C/c1noc(-c2ccccc2)n1)\c1ccccc1
OA25	C(=C/c1ncon1)\c1ccccc1
OA26	NC(=O)[C@@H](Cc1ccccc1)N[SH](=O)=O
OA27	O=C[C@@H](Cc1ccccc1)N[SH](=O)=O
OA28	NC(=O)[C@H](Cc1ccccc1)N[SH](=O)=O
OA29	O=CC1CCN(c2nn3cc(-c4ccc(F)cc4)nc3s2)CC1
OA30	COc1ccc(-c2nc3cc(N)ccc3o2)cc1
OA31	Nc1ccc(-c2nc3ncccc3o2)cc1
OA32	C=Cc1noc(-c2ccccc2O)n1
OA33	C=Cc1ncon1
OA34	C1=C[C@H](C2CCCCC2)CCO1
OA35	O=CC1=C[C@H](C2CCCCC2)C[C@H](O)O1
OA36	O[C@H]1C[C@@H](C2CCCCC2)C=CO1
OA37	C=Cc1noc(-c2ccccc2)n1
OA38	O=CC1=C[C@H](C2CCCCC2)CCO1

Table S12. Statistics related to molecular fragments identified in the active and inactive datasets. In particular, records related to molecular fragments detected exclusively in the active and inactive compounds are highlighted in red. Statistics related to fragments found to be in common between the two classes, but with higher prevalence in the inactives and actives are reported in black and blue, respectively.

Class	Actives (≤500nM)	Inactives (≥ 1μM)	Inactives (≥ 5μM)	Inactives (≥ 10μM)	Inactives (≥ 20μM)	Common Inactives (≥ 1μM) / Active	Common Inactives (≥ 5μM) / Active	Common Inactives (≥ 10μM) / Active	Common Inactives (≥ 20μM) / Active	Common Active / Inactives (≥ 1μM)	Common Active / Inactives (≥ 5μM)	Common Active / Inactives (≥ 10μM)	Common Active / Inactives (≥ 20μM)
Number of fragments	38	70330	63678	57407	25637	44	50	52	63	1658	1604	1567	1303
% Of aromatic fragments	52.6	74.8	74.0	74.3	71.5	50.0	52.0	50.0	47.6	68.0	66.9	67.5	69.8
% Of heteroaromatic fragments	42.1	45.8	45.3	45.5	42.9	40.9	42.0	42.3	36.5	28.0	26.0	26.2	24.1
% Of aliphatic ring fragments	55.3	39.9	40.3	40.0	38.5	25.0	28.0	26.9	27.0	21.2	21.6	20.5	18.5
% Of hetero aliphatic ring fragments	52.6	34.3	34.5	34.4	33.5	20.5	24.0	23.1	20.6	20.3	20.7	19.5	17.4
% Of saturated ring fragments	23.7	17.0	17.4	17.1	16.0	13.6	16.0	15.4	15.9	6.9	7.0	7.1	7.4
% Of HBA fragments	100.0	96.8	96.7	96.6	95.9	84.1	84.0	82.7	74.6	94.9	94.8	94.7	94.2
% Of amide fragments	10.5	27.4	27.5	27.6	28.6	0.0	0.0	0.0	0.0	32.1	32.5	32.7	30.0
FractionCSP3	0.4	0.3	0.3	0.3	0.3	0.3	0.3	0.3	0.4	0.3	0.3	0.3	0.3

Table S13. Molecular fragments that provided the larger difference in their prevalence across the active and inactive classes. Molecular fragments that resulted most prevalently present in the inactive compounds are reported in blue. Molecular fragments that provided a difference in their prevalence across the active and inactive classes lower than 1% are not reported.

SMILES	*Percentage of actives (≤ 500nM)*	*Percentage of inactives (≥ 1μM)*	*Percentage of inactives (≥ 5μM)*	*Percentage of inactives (≥ 10μM)*	*Percentage of inactives (≥ 20μM)*
C=O	36.1	26.7	26.3	26.3	27.7
CN	21.1	12.7	12.1	12.0	12.2
Nc1ccccc1	10.1	3.0	2.8	2.7	2.6
Cc1ccccc1	17.1	11.0	11.0	11.1	11.6
COc1ccccc1	10.1	5.0	5.0	5.0	4.8
CC=O	23.6	18.8	19.1	19.3	20.7
CC(=O)Nc1ccccc1	5.3	0.8	0.8	0.7	0.7
CO	30.4	26.0	26.0	26.1	26.8
CCOc1ccccc1	5.4	1.1	1.1	1.1	0.9
NCCO	5.2	1.2	1.0	0.9	0.9
CC	24.3	20.3	20.5	20.9	21.8
O=Cc1ccccc1	6.5	2.7	2.7	2.7	2.8
O=CNc1ccccc1	3.9	0.2	0.2	0.2	0.3
Oc1ccccc1	6.2	2.7	2.7	2.7	2.6
c1ccc2ocnc2c1	4.1	0.6	0.5	0.5	0.4
O=C(Nc1ccccc1)c1ccccc1	3.6	0.4	0.4	0.4	0.4
CCO	12.0	9.0	9.0	9.2	9.1
c1ccc2scnc2c1	5.1	2.1	1.8	1.7	1.5
COc1ccc(C)cc1	3.6	0.7	0.7	0.7	0.8
NC(=O)c1ccccc1	3.1	0.2	0.2	0.2	0.3
Clc1ccccc1	10.1	7.2	7.2	7.2	7.4
COc1ccc(C=O)cc1	3.2	0.4	0.4	0.4	0.4
c1cnoc1	3.8	1.0	1.0	1.1	1.2
CCc1ccccc1	3.5	0.9	0.9	0.9	1.1
O=CCOc1ccccc1	2.9	0.3	0.3	0.3	0.3
Cc1cccc(O)c1	2.9	0.3	0.3	0.3	0.3
Cc1ccc(O)cc1	2.9	0.4	0.4	0.4	0.3
COc1cccc(C)c1	2.9	0.5	0.5	0.6	0.6
N=Cc1ccccc1	2.5	0.3	0.3	0.3	0.2
[H]/N=C/c1ccccc1	2.3	0.1	0.1	0.1	0.1
Cc1ccc(N)cc1	2.5	0.4	0.4	0.4	0.4
CNC(C)=O	2.1	0.1	0.1	0.1	0.2
COc1ccc(C(N)=O)cc1	2.1	0.1	0.1	0.1	0.1
c1ccc(-c2nc3ccccc3o2)cc1	2.1	0.1	0.1	0.1	0.0
CCNC	3.8	1.8	1.8	1.8	2.0
COc1ccccc1O	2.3	0.3	0.3	0.3	0.3
Nc1cccc(C=O)c1	2.2	0.3	0.3	0.3	0.3
COC	2.5	0.6	0.5	0.5	0.6
NCc1ccccc1	2.3	0.4	0.4	0.4	0.5

NC(=O)COc1ccccc1	2.0	0.1	0.1	0.1	0.1
O=C([O-])c1ccccc1	2.0	0.1	0.1	0.1	0.1
Cc1cccc(N)c1	2.1	0.3	0.3	0.3	0.4
Nc1ccc(O)cc1	2.0	0.2	0.2	0.2	0.2
CCc1ccc(OC)cc1	2.0	0.2	0.2	0.2	0.2
COc1ccc(N)cc1	2.5	0.7	0.7	0.7	0.6
CC(N)=O	3.1	1.3	1.4	1.3	1.4
CCOc1ccc(C=O)cc1	1.9	0.1	0.1	0.1	0.1
CNC=O	1.9	0.1	0.1	0.1	0.1
Nc1ccc2ocnc2c1	1.9	0.1	0.1	0.1	0.0
CCCC	3.3	1.5	1.6	1.6	1.9
O=[NH+][O-]	5	9.7	9.5	9.3	7.7
c1ccoc1	5.5	9.4	9.8	10	10.9
C#N	2.2	4.8	5	5.1	5.7
C1COCCN1	1	3.4	3.6	3.6	3.8
C1CNCCN1	5.9	8	8.2	8.3	8.2
c1ccc2[nH]ccc2c1	1.4	3.4	3.5	3.5	3.5
c1cscn1	1.8	3.5	3.3	3.1	2.4
Cc1ccco1	1.8	3.4	3.6	3.8	4.2

Table S14. Combinations of fragments more frequently observed in the active ligands, according to percentages of occurrence. Sections A and B report the top-100 combinations of 2 and 3 fragments, respectively.

Section A

Fragment #1 (SMILES)	Fragment #2 (SMILES)	Fragment combination (% of Actives)	Fragment #1 (% of Actives)	Fragment #2 (% of Actives)
C=O	c1ccccc1	31.7	36.1	70.7
CO	c1ccccc1	25.7	30.4	70.7
CC	c1ccccc1	20.0	24.3	70.7
CC=O	c1ccccc1	19.8	23.6	70.7
CN	c1ccccc1	19.4	21.1	70.7
C=O	CN	17.9	36.1	21.1
Cc1ccccc1	c1ccccc1	15.8	17.1	70.7
C=O	CO	14.0	36.1	30.4
C=O	CC	12.0	36.1	24.3
CC	CCO	11.0	24.3	12.0
CCO	c1ccccc1	10.4	12.0	70.7
Nc1ccccc1	c1ccccc1	9.3	10.1	70.7
Clc1ccccc1	c1ccccc1	9.2	10.1	70.7
COc1ccccc1	c1ccccc1	9.1	10.1	70.7
CO	COc1ccccc1	7.5	30.4	10.1
C=O	CCO	7.1	36.1	12.0
CC=O	CO	7.1	23.6	30.4
CC	CN	6.6	24.3	21.1
CC	CC=O	6.5	24.3	23.6
CN	CO	6.3	21.1	30.4
CC	CO	6.2	24.3	30.4
C=O	Cc1ccccc1	5.9	36.1	17.1
O=Cc1ccccc1	c1ccccc1	5.9	6.5	70.7
Fc1ccccc1	c1ccccc1	5.7	6.2	70.7
C=O	CC=O	5.7	36.1	23.6
Oc1ccccc1	c1ccccc1	5.6	6.2	70.7
C1CNCCN1	c1ccccc1	5.3	5.9	70.7
CC=O	Cc1ccccc1	5.2	23.6	17.1
CCC	c1ccccc1	5.0	7.0	70.7
C=O	Nc1ccccc1	4.9	36.1	10.1
CCOc1ccccc1	c1ccccc1	4.9	5.4	70.7
c1ccccc1	c1ccncc1	4.9	70.7	7.0
NCCO	c1ccccc1	4.8	5.2	70.7
C=O	O=Cc1ccccc1	4.8	36.1	6.5
c1ccccc1	c1ccoc1	4.8	70.7	5.5
CC(=O)Nc1ccccc1	c1ccccc1	4.8	5.3	70.7
CO	Cc1ccccc1	4.8	30.4	17.1
C=O	Clc1ccccc1	4.8	36.1	10.1
CNC	c1ccccc1	4.6	4.8	70.7
O=[NH+][O-]	c1ccccc1	4.6	5.0	70.7
NCCO	CC=O	4.5	5.2	23.6

CC	Cc1ccccc1	4.5	24.3	17.1
CN	Nc1ccccc1	4.4	21.1	10.1
CC	CCOc1ccccc1	4.2	24.3	5.4
CCO	CCOc1ccccc1	4.2	12.0	5.4
CCO	CN	4.2	12.0	21.1
O=[N+]([O-])c1ccccc1	c1ccccc1	4.1	4.3	70.7
C=O	COc1ccccc1	4.0	36.1	10.1
CC(=O)Nc1ccccc1	CC=O	4.0	5.3	23.6
O=[NH+][O-]	O=[N+]([O-])c1ccccc1	3.8	5.0	4.3
CC=O	Nc1ccccc1	3.6	23.6	10.1
CC(=O)Nc1ccccc1	Nc1ccccc1	3.6	5.3	10.1
CCNC	c1ccccc1	3.5	3.8	70.7
c1ccc2ocnc2c1	c1ccccc1	3.5	4.1	70.7
c1ccc2scnc2c1	c1ccccc1	3.5	5.1	70.7
c1ccccc1	c1cnoc1	3.5	70.7	3.8
CCO	CCOC=O	3.5	12.0	3.5
CCN	c1ccccc1	3.4	4.1	70.7
O=CNc1ccccc1	c1ccccc1	3.4	3.9	70.7
C=O	CCC	3.4	36.1	7.0
C=O	CNC	3.4	36.1	4.8
CC	CCOC=O	3.3	24.3	3.5
C1CCNCC1	c1ccccc1	3.3	4.3	70.7
CCc1ccccc1	c1ccccc1	3.3	3.5	70.7
C=O	Fc1ccccc1	3.3	36.1	6.2
Nc1ccccc1	O=CNc1ccccc1	3.2	10.1	3.9
C=O	CCOC=O	3.2	36.1	3.5
CN	Cc1ccccc1	3.2	21.1	17.1
CO	Oc1ccccc1	3.2	30.4	6.2
c1ccccc1	c1ccsc1	3.2	70.7	4.9
CN	Clc1ccccc1	3.1	21.1	10.1
CO	COC=O	3.1	30.4	3.4
CCC=O	c1ccccc1	3.1	3.9	70.7
CC	CCC	3.0	24.3	7.0
CC	CCNC	3.0	24.3	3.8
O=C(Nc1ccccc1)c1ccccc1	c1ccccc1	3.0	3.6	70.7
CCOC=O	c1ccccc1	3.0	3.5	70.7
C=O	COC=O	2.9	36.1	3.4
C=O	c1ccncc1	2.9	36.1	7.0
COc1ccccc1	Oc1ccccc1	2.9	10.1	6.2
COc1ccc(C)cc1	c1ccccc1	2.9	3.6	70.7
C=O	O=C(Nc1ccccc1)c1ccccc1	2.9	36.1	3.6
C=O	c1ccc2ocnc2c1	2.9	36.1	4.1
NCCS	CC=O	2.9	3.0	23.6
C=NNC	c1ccccc1	2.9	3.4	70.7
C=O	c1ccoc1	2.8	36.1	5.5
CC	COc1ccccc1	2.8	24.3	10.1
CC=O	COc1ccccc1	2.8	23.6	10.1

CC	CCc1ccccc1	2.8	24.3	3.5
CO	O=Cc1ccccc1	2.8	30.4	6.5
COc1ccc(C=O)cc1	c1ccccc1	2.8	3.2	70.7
C1CCNCC1	C=O	2.7	4.3	36.1
CC=O	CCO	2.7	23.6	12.0
C=O	O=CNc1ccccc1	2.7	36.1	3.9
CO	Nc1ccccc1	2.7	30.4	10.1
C=O	CCNC	2.7	36.1	3.8
COC=O	c1ccccc1	2.5	3.4	70.7
C=O	COc1ccc(C=O)cc1	2.5	36.1	3.2
Cc1ccccc1	Nc1ccccc1	2.5	17.1	10.1
NC(=O)c1ccccc1	O=Cc1ccccc1	2.5	3.1	6.5

Section B

Fragment #1 (SMILES)	Fragment #2(SMILES)	Fragment #3 (SMILES)	Fragment combination (% of Actives)	Fragment #1 (% of Actives)	Fragment #2 (% of Actives)	Fragment #3 (% of Actives)
C=O	CN	c1ccccc1	16.0	36.1	21.1	70.7
C=O	CO	c1ccccc1	14.0	36.1	30.4	70.7
C=O	CC	c1ccccc1	9.3	36.1	24.3	70.7
CC	CCO	c1ccccc1	9.1	24.3	12.0	70.7
CO	COc1ccccc1	c1ccccc1	8.4	30.4	10.1	70.7
C=O	CC	CCO	6.1	36.1	24.3	12.0
CC=O	CO	c1ccccc1	6.0	23.6	30.4	70.7
C=O	O=Cc1ccccc1	c1ccccc1	6.0	36.1	6.5	70.7
C=O	CCO	c1ccccc1	5.9	36.1	12.0	70.7
CN	CO	c1ccccc1	5.6	21.1	30.4	70.7
CC	CO	c1ccccc1	5.5	24.3	30.4	70.7
C=O	CN	CO	5.4	36.1	21.1	30.4
C=O	CC	CN	5.4	36.1	24.3	21.1
C=O	COc1ccccc1	c1ccccc1	5.4	36.1	10.1	70.7
NCCO	CC=O	c1ccccc1	5.4	5.2	23.6	70.7
CC	CN	c1ccccc1	5.3	24.3	21.1	70.7
C=O	Clc1ccccc1	c1ccccc1	5.1	36.1	10.1	70.7
CC=O	Cc1ccccc1	c1ccccc1	5.1	23.6	17.1	70.7
CC	CC=O	c1ccccc1	4.9	24.3	23.6	70.7
C=O	CC=O	c1ccccc1	4.8	36.1	23.6	70.7
C=O	Cc1ccccc1	c1ccccc1	4.5	36.1	17.1	70.7
C=O	CO	COc1ccccc1	4.1	36.1	30.4	10.1
CC	CCOc1ccccc1	c1ccccc1	4.1	24.3	5.4	70.7
O=[NH+][O-]	O=[N+]([O-])c1ccccc1	c1ccccc1	4.1	5.0	4.3	70.7
C=O	Nc1ccccc1	c1ccccc1	4.0	36.1	10.1	70.7
CC	CCO	CCOc1ccccc1	4.0	24.3	12.0	5.4
CCO	CCOc1ccccc1	c1ccccc1	4.0	12.0	5.4	70.7
CC(=O)Nc1ccccc1	CC=O	c1ccccc1	3.8	5.3	23.6	70.7
C=O	CNC	c1ccccc1	3.8	36.1	4.8	70.7
CC	Cc1ccccc1	c1ccccc1	3.6	24.3	17.1	70.7

CN	Nc1ccccc1	c1ccccc1	3.5	21.1	10.1	70.7
C=O	CCO	CN	3.5	36.1	12.0	21.1
CC=O	Nc1ccccc1	c1ccccc1	3.5	23.6	10.1	70.7
CO	Cc1ccccc1	c1ccccc1	3.5	30.4	17.1	70.7
CC(=O)Nc1ccccc1	CC=O	Nc1ccccc1	3.4	5.3	23.6	10.1
CC	CCO	CN	3.4	24.3	12.0	21.1
C=O	COc1ccc(C=O)cc1	c1ccccc1	3.4	36.1	3.2	70.7
CC=O	Clc1ccccc1	c1ccccc1	3.4	23.6	10.1	70.7
CC(=O)Nc1ccccc1	Nc1ccccc1	c1ccccc1	3.4	5.3	10.1	70.7
CO	O=Cc1ccccc1	c1ccccc1	3.3	30.4	6.5	70.7
C=O	CN	Nc1ccccc1	3.3	36.1	21.1	10.1
CO	Oc1ccccc1	c1ccccc1	3.2	30.4	6.2	70.7
CCO	CN	c1ccccc1	3.2	12.0	21.1	70.7
CN	Clc1ccccc1	c1ccccc1	3.2	21.1	10.1	70.7
CC=O	O=CCOc1ccccc1	c1ccccc1	3.1	23.6	2.9	70.7
C=O	c1ccccc1	c1ccoc1	3.1	36.1	70.7	5.5
C=O	CC	CO	3.0	36.1	24.3	30.4
C=O	CN	Clc1ccccc1	3.0	36.1	21.1	10.1
O=Cc1ccco1	c1ccccc1	c1ccoc1	3.0	2.7	70.7	5.5
CC	COc1ccccc1	c1ccccc1	3.0	24.3	10.1	70.7
C=O	CO	O=Cc1ccccc1	3.0	36.1	30.4	6.5
C=O	NC(=O)c1ccccc1	c1ccccc1	3.0	36.1	3.1	70.7
C=O	c1ccccc1	c1cnoc1	2.9	36.1	70.7	3.8
C=O	CO	COC=O	2.8	36.1	30.4	3.4
C=O	O=C(Nc1ccccc1)c1ccccc1	c1ccccc1	2.8	36.1	3.6	70.7
COc1ccccc1	Oc1ccccc1	c1ccccc1	2.8	10.1	6.2	70.7
NCCO	Cc1ccccc1	c1ccccc1	2.8	5.2	17.1	70.7
NCCO	CC=O	Cc1ccccc1	2.8	5.2	23.6	17.1
C=O	CO	COc1ccc(C=O)cc1	2.7	36.1	30.4	3.2
NC(=O)c1ccccc1	O=Cc1ccccc1	c1ccccc1	2.7	3.1	6.5	70.7
CO	COc1ccc(C=O)cc1	c1ccccc1	2.7	30.4	3.2	70.7
CC	CCc1ccccc1	c1ccccc1	2.7	24.3	3.5	70.7
CC	CCO	CCOC=O	2.7	24.3	12.0	3.5
C=O	CCO	CCOC=O	2.5	36.1	12.0	3.5
NCCO	CC(=O)Nc1ccccc1	CC=O	2.5	5.2	5.3	23.6
C=O	COC=O	c1ccccc1	2.5	36.1	3.4	70.7
CO	COc1ccc(C)cc1	c1ccccc1	2.5	30.4	3.6	70.7
NCCO	CC(=O)Nc1ccccc1	c1ccccc1	2.5	5.2	5.3	70.7
CC	COc1ccc(C)cc1	c1ccccc1	2.5	24.3	3.6	70.7
C=O	c1ccccc1	c1ccncc1	2.4	36.1	70.7	7.0
CO	COC=O	c1ccccc1	2.4	30.4	3.4	70.7
CC	COc1ccc(CCN)cc1	c1ccccc1	2.4	24.3	1.4	70.7
CC=O	COc1ccccc1	c1ccccc1	2.4	23.6	10.1	70.7

CC	CCc1ccc(OC)cc1	c1ccccc1	2.4	24.3	2.0	70.7
C=O	CCNC	c1ccccc1	2.4	36.1	3.8	70.7
C=O	Fc1ccccc1	c1ccccc1	2.4	36.1	6.2	70.7
C=O	CC	CCOC=O	2.4	36.1	24.3	3.5
C=O	NC(=O)c1ccccc1	O=Cc1ccccc1	2.4	36.1	3.1	6.5
CC	CCNC	c1ccccc1	2.4	24.3	3.8	70.7
C=O	CCOc1ccccc1	c1ccccc1	2.3	36.1	5.4	70.7
CN	COc1ccccc1	c1ccccc1	2.3	21.1	10.1	70.7
CCO	CCOC=O	c1ccccc1	2.3	12.0	3.5	70.7
NCCO	CC(=O)Nc1ccccc1	Nc1ccccc1	2.3	5.2	5.3	10.1
C=O	c1ccc2ocnc2c1	c1ccccc1	2.3	36.1	4.1	70.7
NCCO	Nc1ccccc1	c1ccccc1	2.3	5.2	10.1	70.7
NCCO	CC=O	Nc1ccccc1	2.3	5.2	23.6	10.1
C=O	O=Cc1ccco1	c1ccccc1	2.2	36.1	2.7	70.7
C=O	CC	CCNC	2.2	36.1	24.3	3.8
C=O	CCOC=O	c1ccccc1	2.2	36.1	3.5	70.7
C1CNCCN1	C=O	c1ccccc1	2.2	5.9	36.1	70.7
CO	Clc1ccccc1	c1ccccc1	2.2	30.4	10.1	70.7
NCCO	O=CCOc1ccccc1	c1ccccc1	2.2	5.2	2.9	70.7
Cc1cccc(O)c1	COc1cccc(C)c1	c1ccccc1	2.2	2.9	2.9	70.7
C=O	CN	O=C(Nc1ccccc1)c1cccc c1	2.2	36.1	21.1	3.6
COc1ccccc1O	COc1ccccc1OC	c1ccccc1	2.2	2.3	2.5	70.7
CNC	CO	c1ccccc1	2.2	4.8	30.4	70.7
CN	O=C(Nc1ccccc1)c1cccc c1	c1ccccc1	2.2	21.1	3.6	70.7
CN	O=Cc1ccccc1	c1ccccc1	2.2	21.1	6.5	70.7
CC	CC=O	CCO	2.2	24.3	23.6	12.0
NCCO	CC=O	O=CCOc1ccccc1	2.2	5.2	23.6	2.9

Ligand Clustering

Cluster analyses were performed to evaluate whether the results observed in the ECFP4*fp*-based estimations could derive from a high number of small subsets of structurally close compounds, or from common structural patterns in the entire dataset. To this aim, different datasets including active (*Potency* ≤ 500 nM) and inactive ligands with activity values equal or higher than 1 μM, 5 μM, 10 μM and 20 μM were generated. Then, the datasets were clustered by using the *Canvas* utility available in the Schrödingersuite 2020-1 [1]. In particular, the ECFP4*fp* similarities between active and inactive ligands were first processed to generate a series of pairwise matrices (one for each dataset). Then, generate matrices were processed with the *canvasHC* utility [1], which allows to perform full hierarchical clustering. Defaults settings were used to calculate distances between the groups of ligands (linkage method: "Average"), while the number of clusters was identified by the minimum in the Kelley cost function [2], which is the default option for this software. As shown in **Table A**, the number of clusters varied according to the dataset of compounds investigated in the analyses, heavily increasing with higher threshold of inactivity. To some extent, this suggests the presence of different chemical patterns between active and inactive molecules, which, however, are more evident with the increase of the threshold of inactivity.

Table 4. *fp* similarity records. Finally, the similarity records for each cluster were analyzed, to identify the active compounds providing the best prediction performances in terms of AUC and EF (see **Table S7**).

Dataset	*Number ofcompounds*	*Number ofclusters*	*Number of compoundsin the biggest cluster*	*Number ofsingletones*
A I_5μM	41190	4	41185	2
A I_1μM	48096	4	48091	2
A I_10μM	36316	8587	102	2607
A I_20μM	16915	5549	62	2041

References

1. Schrödinger Release 2020-1: Canvas, Schrödinger, LLC, New York, NY, 2020.
2. Kelley, L.A.; Gardner, S.P.; Sutcliffe, M.J. An automated approach for clustering an ensemble of NMR-derived protein structures into conformationally related subfamilies. *Protein Eng.* **1996**, *9(11)*,1063-1065, doi: 10.1093/protein/9.11.1063.
